# An epilepsy-associated K_V_1.2 charge-transfer-center mutation impairs K_V_1.2 and K_V_1.4 trafficking

**DOI:** 10.1073/pnas.2113675119

**Published:** 2022-04-19

**Authors:** Michelle Nilsson, Sarah H. Lindström, Maki Kaneko, Kaiqian Wang, Teresa Minguez-Viñas, Marina Angelini, Federica Steccanella, Deborah Holder, Michela Ottolia, Riccardo Olcese, Antonios Pantazis

**Affiliations:** ^a^Division of Neurobiology, Department of Biomedical and Clinical Sciences (BKV), Linköping University, 581 83 Linköping, Sweden;; ^b^Center for Personalized Medicine, Children's Hospital Los Angeles, Los Angeles, CA 90027;; ^c^Division of Genomic Medicine, Department of Pathology, Children's Hospital Los Angeles, Los Angeles, CA 90027;; ^d^Division of Molecular Medicine, Department of Anesthesiology & Perioperative Medicine, David Geffen School of Medicine, University of California, Los Angeles, CA 90095;; ^e^Comprehensive Epilepsy Program, Children's Hospital Los Angeles, Los Angeles, CA 90027;; ^f^UCLA Cardiovascular Theme, David Geffen School of Medicine, University of California, Los Angeles, CA 90095;; ^g^Brain Research Institute, David Geffen School of Medicine, University of California, Los Angeles, CA 90095;; ^h^Department of Physiology, David Geffen School of Medicine, University of California, Los Angeles, CA 90095;; ^i^Wallenberg Center for Molecular Medicine, Linköping University, 581 83 Linköping, Sweden

**Keywords:** ion channel, channelopathy, fluorometry, trafficking, dominant negative

## Abstract

We report on a heterozygous *KCNA2* variant in a child with epilepsy. *KCNA2* encodes K_V_1.2 subunits, which form homotetrameric potassium channels and participate in heterotetrameric channel complexes with other K_V_1-family subunits, regulating neuronal excitability. The mutation causes substitution F233S at the K_V_1.2 charge transfer center of the voltage-sensing domain. Immunocytochemical trafficking assays showed that K_V_1.2(F233S) subunits are trafficking deficient and reduce the surface expression of wild-type K_V_1.2 and K_V_1.4: a dominant-negative phenotype extending beyond *KCNA2*, likely profoundly perturbing electrical signaling. Yet some K_V_1.2(F233S) trafficking was rescued by wild-type K_V_1.2 and K_V_1.4 subunits, likely in permissible heterotetrameric stoichiometries: electrophysiological studies utilizing applied transcriptomics and concatemer constructs support that up to one or two K_V_1.2(F233S) subunits can participate in trafficking-capable heterotetramers with wild-type K_V_1.2 or K_V_1.4, respectively, and that both early and late events along the biosynthesis and secretion pathway impair trafficking. These studies suggested that F233S causes a depolarizing shift of ∼48 mV on K_V_1.2 voltage dependence. Optical tracking of the K_V_1.2(F233S) voltage-sensing domain (rescued by wild-type K_V_1.2 or K_V_1.4) revealed that it operates with modestly perturbed voltage dependence and retains pore coupling, evidenced by off-charge immobilization. The equivalent mutation in the Shaker K^+^ channel (F290S) was reported to modestly affect trafficking and strongly affect function: an ∼80-mV depolarizing shift, disrupted voltage sensor activation and pore coupling. Our work exposes the multigenic, molecular etiology of a variant associated with epilepsy and reveals that charge-transfer-center disruption has different effects in K_V_1.2 and Shaker, the archetypes for potassium channel structure and function.

## Significance

A child with epilepsy has a previously unreported, heterozygous mutation in *KCNA2*, the gene encoding K_V_1.2 proteins. Four K_V_1.2 assemble into a potassium-selective channel, a protein complex at the neuronal cell surface regulating electrical signaling. K_V_1.2 subunits assemble with other K_V_1-family members to form heterotetrameric channels, contributing to neuronal potassium-channel diversity. The most striking consequence of this mutation is preventing K_V_1.2-subunit trafficking, i.e., their ability to reach the cell surface. Moreover, the mutation is dominant negative, as mutant subunits can assemble with wild-type K_V_1.2 and K_V_1.4, trapping them into nontrafficking heterotetramers and decreasing their functional expression. Thus, K_V_1-family genes’ ability to form heterotetrameric channels is a double-edged sword, rendering K_V_1-family members vulnerable to dominant-negative mutations in a single member gene.

A child patient with epileptic seizures has a de novo, missense, heterozygous mutation c.698T > C in *KCNA2*. *KCNA2* encodes K_V_1.2 voltage-gated potassium channel subunits (1), which are expressed in central neurons and primarily localized to axon initial segments, juxtaparanodes, and axon preterminals (2). K_V_1.2 channels form a delayed rectifier conductance that regulates action potential repolarization, contributing to firing frequency and synaptic transmission (2–4). Neuronal firing behavior can be further modulated by K_V_1.2-subunit interaction with other K_V_1 subunits: e.g., an A-type conductance is formed by K_V_1.2 and K_V_1.4 heteromeric channels (3, 5, 6). A growing number of both gain- and loss-of-function *KCNA2* variants are implicated in epileptic encephalopathy (7–10).

**Fig. 1.**
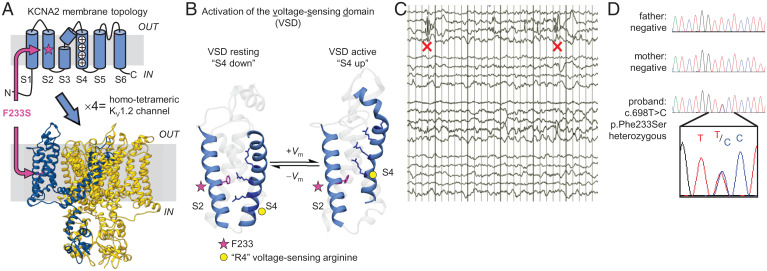
Discovery of a *KCNA2* mutation in a patient with epilepsy. (*A*) Membrane topology of the K_V_1.2 subunit and model of a homotetrameric K_V_1.2 channel (8). F233S is on helix S2, within the VSD. (*B*) Model of K_V_1.2 VSD activation and deactivation (8). The electric field is focused on F233 (magenta, star symbol), which is part of the charge transfer center (11). Upon membrane depolarization (+*V*_m_), positively charged residues on S4 [side chains in dark blue (12)], traverse the membrane electric field, compelling S4 to move outward. Movement of the fourth conserved arginine R4 is thought to couple VSD activation to pore opening (13, 14). Upon membrane repolarization (−*V*_m_), S4 returns to its resting state, inducing pore closure (15–19). (*C*) Partial EEG of the sleeping patient, showing right posterior epileptiform discharges (red X). (*D*) Sequencing chromatograms of the patient and his healthy parents. *Inset*: Magnified view at the mutation site (*KCNA2* c.698T > C, heterozygous).

The mutation causes substitution F233S at the second (S2) transmembrane helix of K_V_1.2 (Fig. 1*A*). Located within the voltage-sensing domain (VSD), F233 is part of the charge transfer center critical for voltage sensitivity (Fig. 1*B*) (11, 13, 20). The equivalent mutation (F290S) in the Shaker K_V_ channel from *Drosophila* impairs voltage-dependent channel opening (11) by disrupting the final VSD activation transition, which couples VSD activation to pore opening (13). However, no investigation of the same mutation has been published in the K_V_1.2 channel, even though the K_V_1.2-2.1 channel chimera (which encompasses the K_V_1.2 F233 site) is the gold standard for potassium channel structure (11, 21). We sought to characterize the effects of mutation F233S in K_V_1.2, as well as its neuronal molecular partner, K_V_1.4.

## Results

### Discovery of a *KCNA2* Variant in a Patient with Epilepsy.

The proband is a male infant with onset, at 15 mo, of a febrile generalized convulsive seizure that lasted 5 min. The second seizure (20 mo), febrile status epilepticus, lasted 45 min. The third seizure (23 mo) was prolonged status epilepticus associated with roseola infection and fever, whereupon he was hospitalized with intubation. The initial electroencephalogram (EEG) showed right hemispheric slowing; a follow-up EEG showed right posterior temporal slowing and multifocal spikes (Fig. 1*C*). The proband continued to have occasional seizures, often associated with illness and fever and usually requiring rescue medication. His development has been delayed with some mild autistic features. The patient was screened with an exome-based DNA panel of 224 epilepsy-associated genes (*SI Appendix*, Table S1). A heterozygous c.698T > C (p.Phe233Ser) missense change in *KCNA2* (transcript: NM_004974.3) was identified. The variant was confirmed in the patient by Sanger sequencing. Targeted Sanger sequencing of DNA from parents showed its de novo origin (Fig. 1*D*). This variant has not been recorded in population databases [the genome aggregation database and the exome aggregation consortium (22), ClinVar (23), and the human gene mutation database (24)] and was classified as potentially deleterious by the Sorting Intolerant from Tolerant (SIFT) (25) and MutationTester2 (26) prediction algorithms. No other pathogenic variants were found.

### F233S Completely Prevents the Cell-Surface Trafficking of K_V_1.2 Channels.

**Fig. 2.**
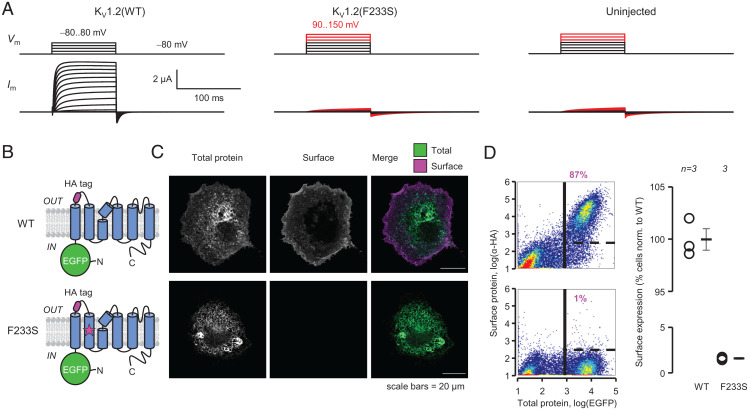
F233S completely prevents the cell-surface trafficking of K_V_1.2 channels. (*A*) Cut-open oocyte Vaseline gap (COVG) experiments showing that *Xenopus* oocytes injected with K_V_1.2(F233S) cRNA exhibit no voltage-dependent currents, even upon strong depolarization (red). *V*_m_, membrane potential; *I*_m_, membrane current. (*B*) Fluorescent K_V_1.2 constructs to investigate trafficking. *Top*: WT K_V_1.2 with N-terminally fused enhanced green fluorescent protein (EGFP), reporting total protein, and extracellularly accessible hemagglutinin (HA) tag, reporting surface protein. *Bottom*: As on top, with F233S. (*C*) Confocal micrographs of nonpermeabilized COS-7 cells transfected with the constructs in *B*. (*D*) Flow cytometry of live COS-7 cells transfected with the constructs in *B*. *Left*: Cell-density plots of log(EGFP) (total protein) against log(α-HA) (cell-surface expression). The vertical solid line separates low- and high-EGFP cells (*Left* and *Right*). The horizontal dashed line separates high-EGFP cells into α-HA negative (*Bottom*) and α-HA positive (*Top*). *Right*: Percentage of α-HA-positive cells, normalized to cells expressing K_V_1.2(WT). Errors are SEM.

Electrophysiological experiments in *Xenopus* oocytes injected with K_V_1.2(F233S) complementary ribonucleic acid (cRNA) revealed no detectable current (Fig. 2*A*). To differentiate between a severe functional or trafficking deficiency, we performed trafficking assays in mammalian COS-7 cells, as previously (8). Cells were transfected with wild-type (WT) or F233S K_V_1.2 constructs (Fig. 2*B*) reporting both total and surface protein (27). These experiments showed that K_V_1.2(F233S) subunits do not traffic to the cell surface (Fig. 2 *C* and *D*).

### K_V_1.2(F233S) Subunits Cause a Dominant-Negative Suppression of K_V_1.2 Channel Conductance.

**Fig. 3.**
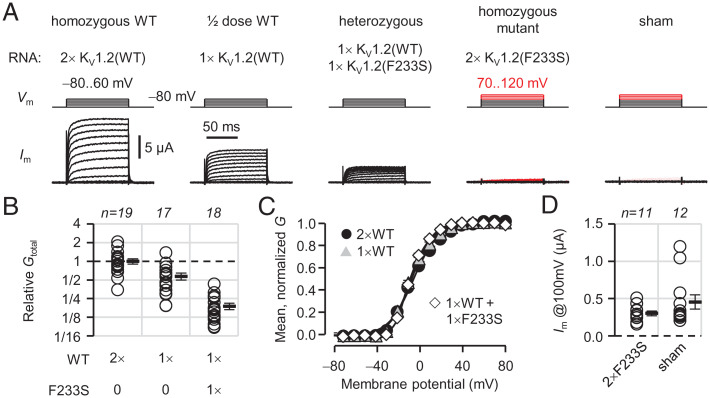
F233S subunits cause a dominant-negative suppression of K_V_1.2 channel conductance. (*A*) Representative OpusXpress (TEVC) current traces from cells injected with K_V_1.2 WT and F233S cRNA. 1×RNA = 0.5 ng/oocyte. (*B*) Macroscopic conductance (*G*_total_) relative to 2× (homozygous) WT (1.0 ± 0.098); 1×WT (0.57 ± 0.073); 1×WT + 1×F233S (0.19 ± 0.022). (*C*) Voltage dependence of 2×WT (*V*_0.5_ = −5.8 ± 0.78 mV; *z*_eff_ = 2.3 ± 0.12 *e*^0^); 1×WT (*V*_0.5_ = −7.6 ± 0.90 mV; *z*_eff_ = 2.7 ± 0.15 *e*^0^); 1×WT + 1×F233S (*V*_0.5_ = −8.3 ± 0.66 mV; *z*_eff_ = 3.3 ± 0.13 *e*^0^). (*D*) Current evoked by depolarization to 100 mV in homozygous mutant (2×F233S; 300 ± 31 nA) and sham-injected cells (450 ± 95 nA); *P* = 0.15. Errors are SEM.

As the patient is heterozygous, both WT and F233S-bearing K_V_1.2 subunits are present in his neurons and could tetramerize during early biogenesis as nascent polypeptides (28). We injected different amounts of WT and F233S cRNA in *Xenopus* oocytes to emulate homozygous WT (double cRNA dose corresponding to two WT alleles), half-dose WT (single cRNA dose corresponding to one WT allele), heterozygous (single WT and single F233S cRNA doses), and homozygous mutant (double F233S cRNA dose). The cells responded linearly to injected cRNA, exhibiting 0.57- ± 0.07-fold conductance in the half-dose WT condition compared to homozygous WT (1.0 ± 0.098) (Fig. 3 *A* and *B*). The former was not significantly different from a theoretical sample with mean 0.5 and the same σ and *n* (*P* = 0.50). Heterozygous cells exhibited ∼20% conductance compared to homozygous-WT cells, significantly less than half-dose WT (*P* = 1.3e−5), demonstrating dominant-negative loss of K_V_1.2 function. The current in these conditions had similar voltage dependence (Fig. 3*C*), indicating that the main effect of F233S is its trafficking defect. Homozygous-mutant cells produced no current (Fig. 3 *A* and *D*).

### K_V_1.2(F233S) Subunits Sequester, and Are Concomitantly Rescued by, K_V_1.2(WT).

**Fig. 4.**
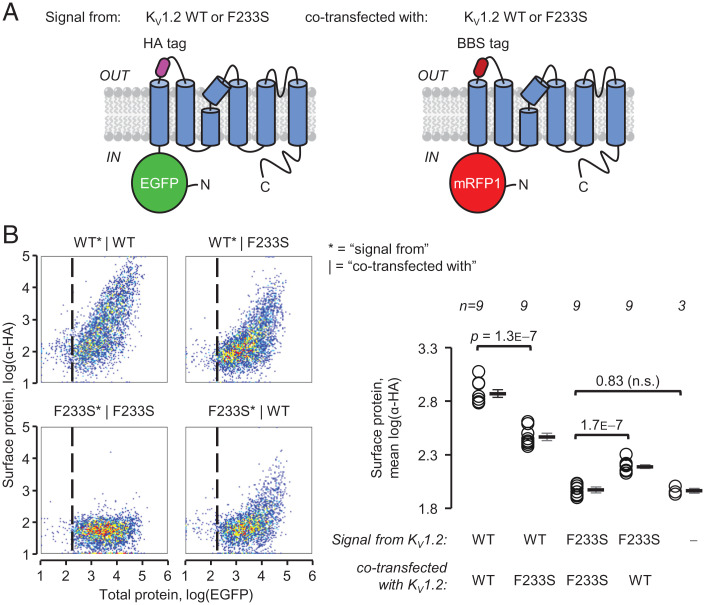
K_V_1.2(F233S) subunits sequester, and are concomitantly rescued by, K_V_1.2(WT). (*A*) Constructs used to evaluate K_V_1.2 cell-surface trafficking. Each construct emulates one allele. K_V_1.2 with N-terminally fused EGFP, reporting total protein production; an extracellular HA tag (as in Fig. 2*B*) cotransfected with K_V_1.2 with N-terminally fused mRFP1, reporting total protein production; and an extracellular bungarotoxin (BTX) binding-site (BBS) tag. (*B*) Flow cytometry experiments on cells transfected with the constructs in *A*. The cell-density plots show total protein [log(EGFP)] and surface staining [log(α-HA)] in live cells positive for mRFP1. The vertical dashed lines separate negative (*Left*) and positive (*Right*) EGFP cells. Percentage of cells with a positive surface (α-HA) signal (normalized to WT*|WT, i.e., 100 ± 0.85%): WT*|F233S: 55 ± 2.0%; F233S*|WT: 22 ± 1.5%; F233S*|F233S: 1.3 ± 0.27%. Signals from mRFP1 and BTX in EGFP-positive cells are in *SI Appendix*, Fig. S1. Errors are SEM. n.s.= not significant.

The dominant-negative loss of K_V_1.2 conductance is likely due to the association of WT and trafficking-deficient F233S subunits, decreasing the availability of the former on the cell surface. We directly evaluated surface trafficking of WT subunits in the presence of either WT or F233S subunits, and vice versa, by flow cytometry. COS-7 cells were transfected with two K_V_1.2 constructs in equal proportion, representing two *KCNA2* alleles, each bearing a different pair of tags reporting total and surface protein expression (Fig. 4*A*). We evaluated surface expression in live cells positive for both total protein tags (Fig. 4*B*). When a K_V_1.2(WT) was coexpressed with another K_V_1.2(WT) construct, it exhibited high surface expression. Crucially, K_V_1.2(WT) trafficking was significantly reduced in the presence of K_V_1.2(F233S).

The previous result suggests that WT and F233S subunits can assemble into trafficking-deficient heterotetramers. Is it possible that a different fraction of WT/F233S heterotetramers can reach the surface? Indeed, a significant amount of F233S subunits were detected on the surface of cells coexpressing WT, indicating rescue (Fig. 4*B*). The same results were obtained using the oppositely labeled constructs, although bungarotoxin signals were weaker than those from hemagglutinin-tagged constructs (*SI Appendix*, Fig. S1).

### Only K_V_1.2(WT/F233S) Heterotetramers with One F233S Subunit Are Likely Trafficking Competent.

**Fig. 5.**
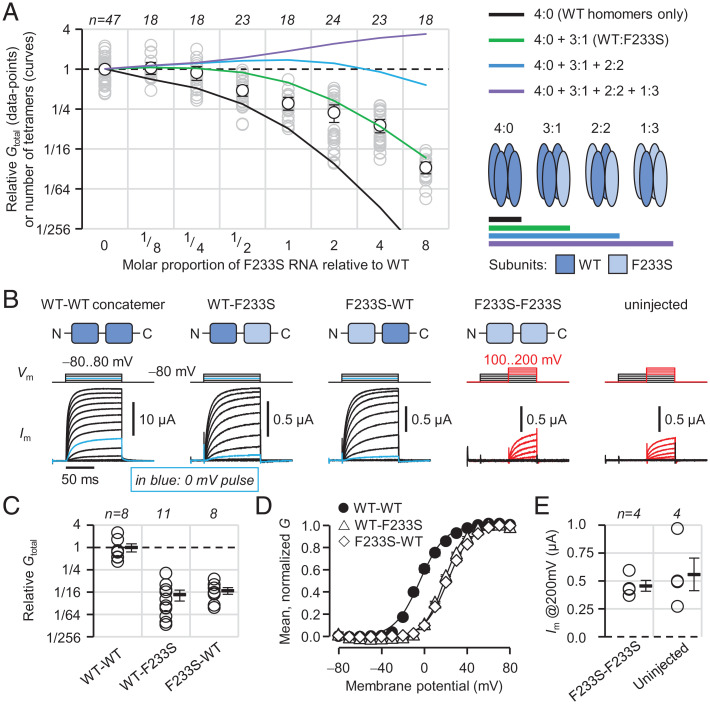
Only 3^WT^:1^F233S^ heterotetramers are trafficking capable. (*A*) Relative conductance in cells injected with K_V_1.2(WT) cRNA and increasing proportion of K_V_1.2(F233S) cRNA. The voltage dependence in all conditions is in *SI Appendix*, Fig. S2. Superimposed curves show number of tetramers of specified composition, relative to the 0-F233S condition, generated by a model assuming binomially distributed tetramerization. (*B*) Representative COVG current traces from cells injected with dimeric K_V_1.2 concatemer cRNA. Relative macroscopic conductance (*C*) and voltage dependence (*D*) of cells injected with K_V_1.2(WT)-K_V_1.2(WT) (WT-WT) cRNA (relative *G*_total_ = 1.0 ± 0.25; *V*_0.5_ = −3.3 ± 1.5 mV; *z*_eff_ = 2.3 ± 0.16*e*^0^), WT-F233S (relative *G*_total_ = 0.053 ± 0.017; *V*_0.5_ = 19 ± 0.29 mV; *z*_eff_ = 2.7 ± 0.083*e*^0^) or F233S-WT (relative *G*_total_ = 0.068 ± 0.014; *V*_0.5_ = 22 ± 0.59 mV; *z*_eff_ = 2.3 ± 0.10*e*^0^). (*E*) No current was observed in cells injected with F233S-F233S up to 200 mV (450 ± 48 nA) compared to uninjected cells (560 ± 150 nA; *P* = 0.53). Errors are 95% CI (*A*) or SEM (*B*–*E*).

The results so far suggest that some K_V_1.2(WT/F233S) heterotetramers are trafficking deficient, causing WT-subunit sequestration, while others are trafficking competent, causing F233S-subunit rescue. Since the N-terminal tetramerization domain of K_V_1 subunits facilitates oligomerization early in biosynthesis (28), one would expect the full complement of tetrameric WT/F233S stoichiometries to form. Could it be that only specific stoichiometries are trafficking competent? To direct the distribution of tetrameric stoichiometries, we implemented an "applied transcriptomics" experimental paradigm, varying the molar proportion of K_V_1.2(F233S) RNA injected in oocytes over a broad range while keeping the amount of WT RNA constant. K_V_1.2 conductance decreased precipitously in a F233S dose-dependent manner (Fig. 5*A*). In cells with the most F233S cRNA, there was also a ∼5-mV depolarizing shift in voltage dependence (*SI Appendix*, Fig. S2 *A* and *B*).

Assuming that F233S does not affect maximal open-probability or single-channel conductance, the data reflect the number of channels on the cell surface. Thus, the data were overlaid with models predicting increasing numbers of F233S subunits participating in trafficking heterotetramers. The model allowing no F233S-containing heterotetramers (black curve) failed to account for the data, predicting too low conductance at high F233S proportions. This is consistent with the observation that F233S subunits are rescued by WT (Fig. 4 and *SI Appendix*, Fig. S1). The green model, allowing one F233S subunit per heterotetramer, described the data best. Models allowing more F233S subunits (cyan and purple curves) greatly overestimated the observed conductance. There was no evidence of cells being overwhelmed by cRNA at the amounts used (*SI Appendix*, Fig. S2 *C*–*E*). A thorough discussion of the model assumptions, considering additional simulations and subsequent experimental results, is included in the *SI Appendix*.

The green model suggested that the 2^WT^:2^F233S^ stoichiometry was trafficking deficient. To corroborate this premise, we constructed concatenated K_V_1.2 dimers, which are expected to assemble as pseudotetrameric dimers of dimers (29, 30). Accordingly, K_V_1.2(WT)-K_V_1.2(F233S) (WT-F233S) dimers exhibited very low conductance (∼5%) compared to WT-WT, supporting that the 2^WT^:2^F233S^ stoichiometry is strongly trafficking impaired (Fig. 5 *B* and *C*). This result independently excluded the cyan model in Fig. 5*A*. Despite the very low expression level of WT-F233S channels, it was possible to characterize their voltage dependence: their half-activation potential was shifted (Δ*V*_0.5_) by 22 ± 0.34 mV compared to WT-WT (Fig. 5*D*), demonstrating that F233S also impaired K_V_1.2 voltage dependence in a dominant-negative manner. The inverted construct F233S-WT had similar properties (Δ*V*_0.5_: 25 ± 0.69 mV; Fig. 5 *B*–*D*). F233S-F233S–injected cells did not exhibit current distinguishable from uninjected cells (Fig. 5 *B* and *E*).

### The K_V_1.2(F233S) Dominant-Negative Trafficking Deficiency Extends to K_V_1.4 Subunits.

**Fig. 6.**
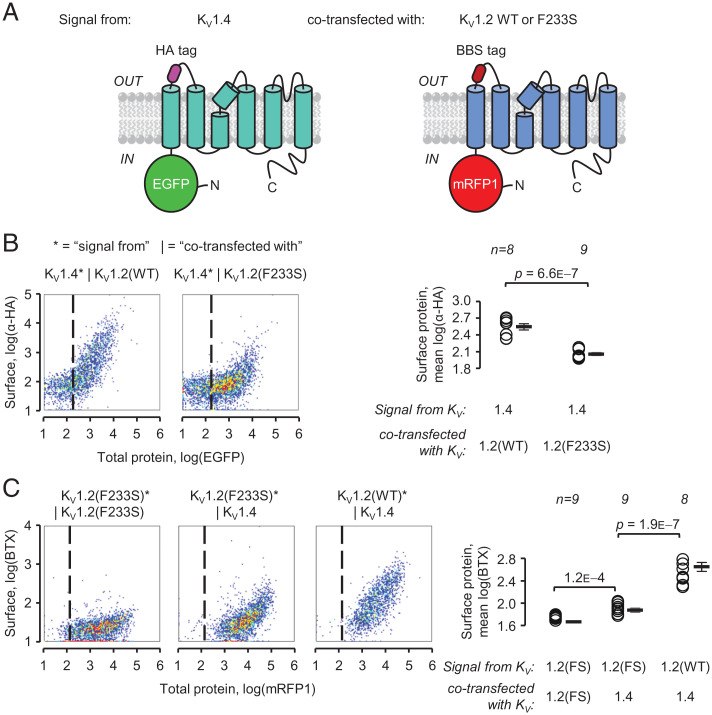
K_V_1.2(F233S) subunits concomitantly suppress and are rescued by K_V_1.4. (*A*) Constructs used to evaluate K_V_1.2 and K_V_1.4 cell-surface trafficking. K_V_1.4 with N-terminally fused EGFP, reporting total protein production; an extracellular HA tag cotransfected with K_V_1.2 with N-terminally fused mRFP1, reporting total protein production; and an extracellular BBS tag. (*B*) Flow cytometry experiments on cells transfected with constructs in *A*. The cell-density plots show total protein [log(EGFP)] and surface staining [log(α-HA)] in live cells positive for mRFP1. The vertical dashed line separates negative (*Left*) and positive (*Right*) EGFP. Percentage of cells with a positive surface (α-HA) signal [normalized to K_V_1.4*|K_V_1.2(WT), i.e., 100 ± 2.4%]: K_V_1.4*|K_V_1.2(F233S): 8.5 ± 1.9%. (*C*) As in *B*, showing the signals from K_V_1.2 constructs cotransfected with K_V_1.4. Plots of log(mRFP1) (total protein) against log(BTX) (cell-surface expression). The vertical dashed line separates negative (*Left*) and positive (*Right*) mRFP1 cells among the plotted live, EGFP-positive cells. Percentage of cells with a positive surface (BTX) signal [normalized to K_V_1.2(WT)*|K_V_1.4, i.e., 100 ± 2.7%]: K_V_1.2(F233S)*|K_V_1.2(F233S): 3.7 ± 0.36%; K_V_1.2(F233S)*|K_V_1.4: 16 ± 3.1%. Errors are SEM.

Paralogous potassium channel subunits were shown to assemble into heteromeric channels soon after their cloning (31, 32). In mammals, K_V_1.4 subunits are molecular partners of K_V_1.2, forming heterotetrameric channels that contribute to the A current in neurons (3, 5, 6). We used flow cytometry to directly evaluate K_V_1.2(F233S) rescue by K_V_1.4 and whether K_V_1.4 subunits are sequestered by K_V_1.2(F233S). COS-7 cells were cotransfected with two constructs: K_V_1.4 and K_V_1.2 (WT or F233S), each with a different set of fluorescent tags for total and surface protein expression (Fig. 6*A*). K_V_1.4 surface trafficking was reduced in the presence of K_V_1.2(F233S) (Fig. 6*B*). At the same time, K_V_1.4 assisted the trafficking of K_V_1.2(F233S) subunits (Fig. 6*C*). This represented partial rescue, as the cells did not achieve the same K_V_1.2 surface levels as for WT subunits (Fig. 6*C*).

### K_V_1.4 and K_V_1.2(F233S) Subunits Likely Form 3:1 and 2:2 Heteromeric Channels.

**Fig. 7.**
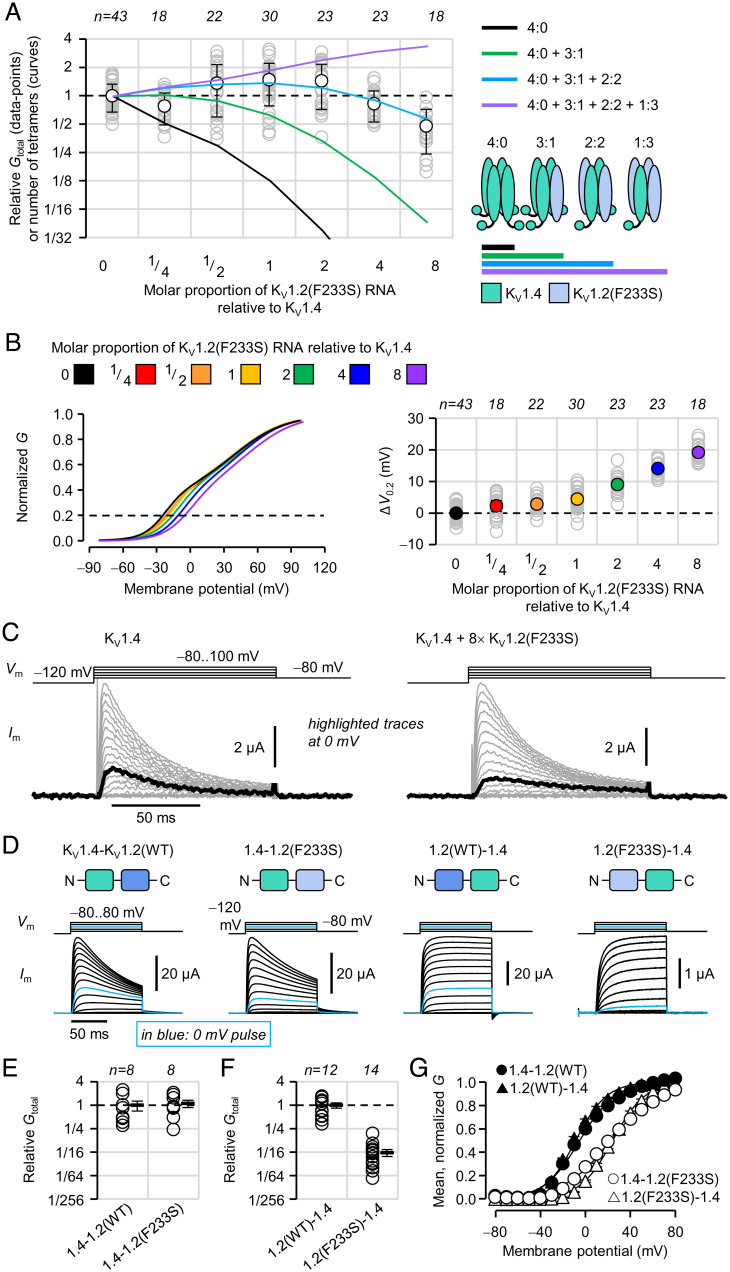
K_V_1.4 and K_V_1.2(F233S) subunits form 3:1 and 2:2 heteromeric channels. (*A*) Relative peak conductance in cells injected with K_V_1.4 cRNA and increasing molar proportion of K_V_1.2(F233S) cRNA. Superimposed curves show the number of tetramers of specified composition relative to the 0-F233S condition, generated by a model assuming binomially distributed tetramerization. (*B*) Fitted Boltzmann distributions of peak macroscopic conductance from the same cells as in *A*. Data, parameters, and individual curves in *SI Appendix*, Fig. S3. The potential of 20% activation (*V*_0.2_) best shows the F233S-dependent shift in voltage dependence on the right. Error bars are ±95% CI. (*C*) Representative TEVC current traces demonstrate altered voltage-dependent properties in heteromeric channels. (*D*) Representative COVG current traces from cells injected with dimeric K_V_1.4/K_V_1.2 concatemer cRNA. Relative macroscopic conductance (*E* and *F*) and voltage dependence (*G*) of cells injected with 1.4-1.2(WT) (relative *G*_total_ = 1.0 ± 0.29; *V*_0.5_ = −4.8 ± 1.3 mV; *z*_eff_ = 1.6 ± 0.038 *e*^0^); 1.4-1.2(F233S) (relative *G*_total_ = 1.1 ± 0.23; *V*_0.5_ = 23 ± 1.2 mV; *z*_eff_ = 1.2 ± 0.027 *e*^0^); 1.2(WT)-1.4 (relative *G*_total_ = 1.0 ± 0.15; *V*_0.5_ = −8.2 ± 0.95 mV; *z*_eff_ = 1.9 ± 0.12 *e*^0^); 1.2(F233S)-1.4 (relative *G*_total_ = 0.062 ± 0.012; *V*_0.5_ = 24 ± 1.2 mV; *z*_eff_ = 1.8 ± 0.044 *e*^0^). Conductance for 1.4-1.2 constructs, which exhibit fast inactivation, was calculated at peak current. Errors are SEM.

K_V_1.4 subunits are more adept at surface trafficking, both lacking endoplasmic reticulum (ER) retention signals and possessing a forward trafficking motif (33–35). In fact, they enhance K_V_1.2 trafficking (36). We hypothesized that K_V_1.4 is more proficient than K_V_1.2(WT) in rescuing K_V_1.2(F233S). Indeed, in cells expressing K_V_1.4, conductance was significantly decreased only when K_V_1.2(F233S) cRNA was added in eightfold excess (Fig. 7*A*). Binomial tetramerization models suggested that K_V_1.4 homotetramers and 3:1 and 2:2 K_V_1.4:K_V_1.2(F233S) heterotetramers can traffic (cyan curve), anticipating also some K_V_1.4 sequestration (no 1:3 heterotetramers). While K_V_1.4 conductance amplitude did not clearly depend on F233S dosage, its voltage dependence did, exhibiting a progressive shift to more depolarized potentials with increasing F233S (Fig. 7 *B* and *C* and *SI Appendix*, Fig. S3) and supporting that K_V_1.4/1.2(F233S) heterotetramers can traffic in oocytes. Note also the spontaneous decrease in current during depolarization (Fig. 7*C*) caused by an N-terminal inactivation motif blocking the channel pore (37, 38).

As before, we used dimeric concatemers to evaluate the trafficking and biophysical properties of the 2:2 K_V_1.4:K_V_1.2 stoichiometry. K_V_1.4-K_V_1.2(F233S) constructs exhibited full conductance compared with K_V_1.4-K_V_1.2(WT) (*P =* 0.76; Fig. 7 *D* and *E*), supporting that the 2:2 K_V_1.4:K_V_1.2(F233S) stoichiometry is trafficking capable. However, the inverted construct, K_V_1.2(F233S)-K_V_1.4, exhibited strongly diminished conductance (Fig. 7 *D* and *F*). The significance of this result for the mechanism of F233S-caused trafficking impairment is discussed below. Both K_V_1.2(F233S)-containing constructs exhibited right-shifted voltage dependence compared to their WT counterparts (Fig. 7*G*). Finally, our constructs with a C-terminal K_V_1.4 lacked fast inactivation (Fig. 7*D*). Since the inactivation particle is located at the K_V_1.4 N terminus (37, 38), perhaps it cannot block the channel when N-terminally tethered.

### Operation of the K_V_1.2(F233S) VSD.

**Fig. 8.**
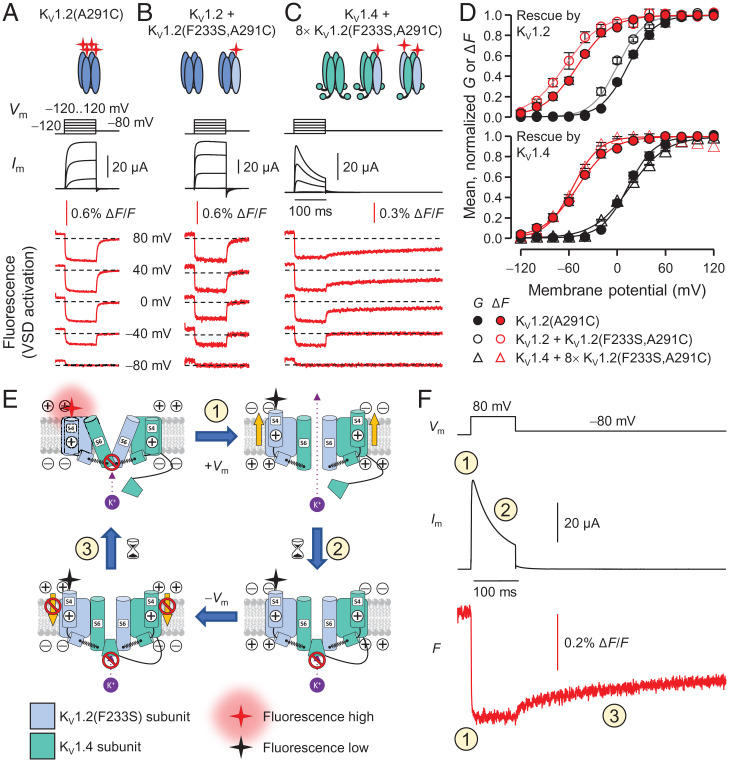
Operation of the K_V_1.2(F233S) VSD. (*A*) VCF experiments on K_V_1.2 homotetramers, fluorescently labeled outside the S4 helix (A291C) to optically track the voltage-dependent activation of the VSD (Fig. 1*B*) *V*_m_, *I*_m_ (black), and simultaneously acquired fluorescence deflections (Δ*F*, red). VSD activation causes fluorophore quenching, reported as negative Δ*F* (8). (*B* and *C*) As in *A*, but the fluorescence label is in K_V_1.2(F233S) subunits rescued by WT K_V_1.2 (*B*) or K_V_1.4 (*C*). Note that VSD deactivation is delayed in *C* (interpreted below). (*D*) Normalized macroscopic conductance (*G*, black) and VSD activation (Δ*F*, red), fit to Boltzmann distributions. K_V_1.2(A291C) *G*: *V*_0.5_ = 14 ± 2.8 mV, *z*_eff_ = 1.6 ± 0.14 *e*^0^; Δ*F*: *V*_0.5_ = −50 ± 1.8 mV, *z*_eff_ = 1.3 ± 0.22 *e*^0^; *n* = 4. K_V_1.2/1.2(F233S,A291C) *G*: *V*_0.5_ = −0.89 ± 2.4 mV, *z*_eff_ = 1.6 ± 0.074 *e*^0^; Δ*F*: *V*_0.5_ = −64 ± 4.0 mV, *z*_eff_ = 1.2 ± 0.11 *e*^0^; *n* = 6. K_V_1.4/1.2(F233S,A291C) *G*: *V*_0.5_ = 16 ± 0.75 mV, *z*_eff_ = 1. 1 ± 0.039 *e*^0^; Δ*F*: *V*_0.5_ = −56 ± 1.0 mV, *z*_eff_ = 1.6 ± 0.13 *e*^0^; *n* = 6. Errors are SEM. Note that the K_V_1.2(A291C) conductance (black filled circles) is right shifted by ∼15 mV compared to WT due to the A291C mutation (8); the K_V_1.2/1.2(F233S,A291C) conductance (black open circles) has a large contribution from WT homotetramers, so it is relatively left shifted. (*E* and *F*) Interpretation of data from K_V_1.4/1.2(F233S,A291C) heterotetramers (*C*). The diagrams in *E* show pore and VSD states in a hypothetical K_V_1.4/1.2(F233S,A291C) heterotetramer (only two subunits shown). Numbered transitions are registered on the exemplary current and fluorescence traces in *F*. (*E*) *Top Left*: The *V*_m_ is negative; the VSDs are resting (label in high-fluorescence state), and the pore is closed (no current). Upon depolarization (transition 1) VSDs activate and the channel opens (*Top Right*), reported as current onset and downward Δ*F* in *F*. Next (transition 2), K_V_1.4 inactivation particles block the channels (*Bottom Right*), reported as current reduction. Following membrane repolarization, the inactivation particle persists in the pore, preventing its closure (*Bottom Left*). It takes time for the particle to dissociate (transition 3), delaying VSD deactivation (off-charge immobilization), reported as slow Δ*F* recovery. In the absence of an inactivation particle, the VSD deactivates with fast kinetics (*SI Appendix*, Fig. S4*B*).

We exploited the rescue of K_V_1.2(F233S) subunits by WT K_V_1.2 and K_V_1.4 to investigate the function of the F233S-bearing VSD. To selectively track the activation of F233S-bearing subunits, we used voltage-clamp fluorometry (VCF) (39, 40). In this approach, a Cys is substituted at a position expected to undergo conformational changes [A291C, at the extracellular flank of K_V_1.2 S4 (8, 41)]. The Cys is then labeled with a thiol-reactive, environment-sensitive fluorophore, such as MTS-TAMRA. Thus, changes in ensemble fluorescence (Δ*F*) report VSD activation transitions. K_V_1.2(A291C) channels showed robust downward Δ*F* signals (Fig. 8*A*), reporting VSD activation upon membrane depolarization and its rapid deactivation upon repolarization. By only incorporating the A291C substitution in F233S-bearing subunits, only the movements of F233S-containing VSDs are optically reported; the VSDs of rescuing K_V_1.2 and K_V_1.4 subunits also undergo voltage-dependent movements, but they are not fluorescently labeled and therefore their movements are not optically reported (*SI Appendix*, Fig. S4). These experiments revealed that the rescued K_V_1.2(F233S) VSD activates with a modestly affected voltage dependence, as well as near-normal valence and activation kinetics (Fig. 8 *A*–*D*).

K_V_1.4 subunits confer a particular advantage to this experimental paradigm, enabling the direct observation of VSD-pore coupling. Their unique (in the K_V_1 family) N-terminal inactivation particle binds to, and blocks, the open pore (*N*-type inactivation, Fig. 7*C*) (37). In doing so, the inactivation particle prevents pore closure (42, 43), which in turn impedes the return of the VSD to its resting conformation. This phenomenon, termed off-charge immobilization, persists until the inactivation particle dissociates and the pore closes. It is experimentally observable in gating-current (44, 45) and optical (46) experiments.

Accordingly, in the presence of K_V_1.4 subunits, and at depolarizations that evoked pore opening (and *N*-type inactivation), K_V_1.2(F233S) VSD deactivation was markedly slower (Fig. 8*C*), consistent with off-charge immobilization. This indicates that the rescued K_V_1.2(F233S) VSDs were functionally coupled to the state of the channel pore (Fig. 8 *E* and *F*). When K_V_1.2(F233S) subunits were rescued by K_V_1.4 lacking an inactivation particle, their VSD deactivated quickly (*SI Appendix*, Fig. S4).

## Discussion

In this work, we report on a *KCNA2* variant discovered in a male child with epilepsy (Fig. 1 *C* and *D*). The protein product of *KCNA2*, K_V_1.2 subunits of voltage-gated potassium channels are expressed throughout the brain as homotetrameric channels (Fig. 1*A*) or participating in heterotetrameric complexes with other K_V_1 channel subunits (47), where they regulate neuronal excitability and synaptic release. The *KCNA2* variant studied here results in the substitution of a residue (F233S) at the core of the bundle of transmembrane helices comprising the channel VSD (Fig. 1*B*). This mutation completely abolished the surface trafficking of K_V_1.2(F233S) subunits (Fig. 2). We also discovered that the mutant subunits are dominant negative, suppressing the surface trafficking of their WT allele (Figs. 3 and 4 and *SI Appendix*, Fig. S1) but also K_V_1.4, a known molecular partner of K_V_1.2 (Fig. 6). Yet the severe trafficking defect of K_V_1.2(F233S) subunits was ameliorated in the presence of WT K_V_1.2 and K_V_1.4 subunits, demonstrated by both surface labeling (Figs. 4 and 6 and *SI Appendix*, Fig. S1) and electrophysiological evidence (Figs. 5 and 7). This allowed the direct observation of the mutant VSD activation (Fig. 8 and *SI Appendix*, Fig. S4). Our work classifies this previously unreported *KCNA2* variant as a dominant-negative mutation that leads to loss of K_V_1.2 channel function as a phenotype and could also decrease the function of other K_V_1-family molecular partners.

### How Does F233S Affect K_V_1.2 Functional Properties?

F233 has an eminent role in voltage sensing as the charge transfer center (Fig. 1*B*) (11). How does its substitution affect channel function? Since K_V_1.2(F233S) homotetramers could not traffic (Fig. 2), it was not possible to directly determine their voltage dependence. In the Shaker K^+^ channel, the archetype for functional K_V_ studies, some VSD mutations have been shown to perturb voltage dependence proportionally to the number of affected subunits (29, 48). K_V_1.2(WT/F233S) concatemers, which represent the 2^WT^:2^F233S^ stoichiometry, exhibit a depolarizing shift (Δ*V*_0.5_) of 24 mV, so we may infer that K_V_1.2(F233S) homotetramers would exhibit a 48-mV shift. The equivalent mutation in Shaker(F290S) induced a stronger Δ*V*_0.5_ of ∼80 mV (11).

The four VSDs in a K_V_ channel are thought to activate independently by membrane depolarization (early transitions) and then undergo a concerted late transition that leads to pore opening (49). In Shaker, mutations that disrupt VSD-pore coupling result in depolarizing shifts of late VSD transitions and pore opening (49), an effect recapitulated by F290S (13). This was not observed in the VSD of K_V_1.2(F233S) (Fig. 8*D*), while off-charge immobilization (in the presence of K_V_1.4) strongly indicates functional VSD-pore coupling (Fig. 8 and *SI Appendix*, Fig. S4). However, the requisite association of K_V_1.2(F233S) with rescuer subunits may have rectified its late VSD transitions, occurring in concert with the movements of WT VSDs in the heterotetrameric channels.

Our view is that charge-transfer-center disruption has similar effects on the two channels, albeit milder in K_V_1.2. Previous work indicates that K_V_1.2 has a thicker hydrophobic septum than Shaker (12), perhaps rendering its VSD more resilient to charge-transfer-center disruption.

### Mechanism of F233S-Mediated Trafficking Impairment.

Why does the cellular protein biosynthesis and secretory machinery prevent K_V_1.2(F233S)-subunit expression? We propose that the F233S mutation hinders trafficking at two distinct stages: "early" and "late". The former likely include processes such as translation, helix formation, and translocation, thought to occur early in channel biosynthesis (50). The latter likely involve the trafficking of translocated subunits and their interaction with ER proteins.

Starting with the K_V_1.4/K_V_1.2(F233S) concatemer experiments, the construct with an N-terminal K_V_1.4 subunit is trafficking capable, whereas the construct with an N-terminal K_V_1.2(F233S) is trafficking impaired (Fig. 7 *D*–*F*). The only difference between the two is the order in which the two domains are synthesized and introduced to the cell secretory pathway. In related K_V_1.3 channels, helix S2 functions as the signal for translocation and establishment of N terminus topology (51). K_V_1.3 S2 helix formation depends on the presence of a WF motif in the middle of the helix (W232 and F233 in K_V_1.2) that prevents premature S2 helical formation (52). As this motif is highly conserved across K_V_ channel subfamilies, its helical-suppressor function is likely crucial to translocation and could be a major first impediment for the functional expression of N-terminal K_V_1.2(F233S) peptides, whether they are monomers (Fig. 2) or concatenated with WT K_V_1.2 (Fig. 5 *B* and *C*) or K_V_1.4 (Fig. 7 *D* and *F*). Aberrant K_V_1.2(F233S) translocation may in turn interfere with other processes important for trafficking, such as electrostatic interactions within the VSD (53) or glycosylation at the S1-S2 linker (54, 55).

By contrast, when K_V_1.2(F233S) is the C-terminal partner of K_V_1.4, trafficking is not affected (Fig. 7 *D* and *E*). In this construct, the N-terminal K_V_1.4 likely undergoes a successful translocation first. Why does the C-terminal K_V_1.2(F233S) not oppose translocation? We suggest that this is evidence of cooperative translocation. It is possible that such a mechanism (a capable N-terminal partner assisting the translocation of downstream domains) is employed by natural concatemers, such as two-pore channels and pseudotetrameric voltage-gated calcium and sodium channels (56).

Why does N-terminal WT K_V_1.2, then, not facilitate the trafficking of C-terminal K_V_1.2(F233S) (Fig. 5 *B* and *C*)? Likely it does, but trafficking is then halted by later events, unrelated to biosynthesis, folding, and translocation. These may include posttranslational modification and interaction with the ER protein quality-control apparatus. K_V_1.2 subunits have ER retention motifs which K_V_1.4 lack; additionally, K_V_1.4 possess forward trafficking signals (33–35). These elements could underlie the successful trafficking of 3:1 and 2:2 K_V_1.4/K_V_1.2(F233S) heterotetramers and K_V_1.4-K_V_1.2(F233S) concatemers, but not 2:2 K_V_1.2(WT)/K_V_1.2(F233S) heterotetramers or the K_V_1.2(WT-F233S) concatemers. It appears that inclusion of trafficking-deficient K_V_1.2(F233S) subunits in heterotetramers imposes a quantal trafficking penalty, abated by trafficking-enhancing elements, as in K_V_1.4.

In conclusion, we propose this model for K_V_1.2(F233S)-subunit trafficking: Nascent peptides tetramerize early (28), assembling into homo- and heterotetramers. K_V_1.2(F233S) homotetramers are immediately excluded, encountering an early translocation impediment and receiving no assistance from WT partner subunits. This early block may also prevent heterotetramers with three K_V_1.2(F233S) subunits from trafficking; alternatively, these are blocked later in the ER. Heterotetramers with two K_V_1.2(F233S) translocate successfully but then have different fates, depending on the trafficking adeptness of their partner subunits: those with K_V_1.2 are blocked (Fig. 5), whereas those with K_V_1.4 traffic successfully (Fig. 7).

By comparison, Shaker(F290S) channels were reported to have "medium current level" (11) and sufficient expression for the measurement of gating currents (13). We find it interesting that while the effects of F→S are similar in the two channels, their severities are inverted: strong functional impairment and modest trafficking deficiency in Shaker, and vice versa for K_V_1.2. This disparity indicates that the trafficking and functional defects are not strictly linked. Since F233/F290 is critical for two altogether different functions—S2-helix formation and translocation of the nascent polypeptide (52) and voltage sensing in the mature protein (11, 13)—these could be differentially modified by other parts of the K_V_1.2 and Shaker channels.

There is still much to discover about the identity of early and late events of K_V_1.2(F233S) trafficking impairment. We are certain that this variant, serendipitously discovered in an epilepsy patient, can serve as a tool to investigate the fundamental aspects of channel biogenesis, trafficking, and oligomerization.

### Neuronal Consequences of a Dominant-Negative KCNA2 Variant.

The ability of K_V_ subunits to form heteromeric channels is a major source of conductance diversity in the brain. However, it can act as a double-edged sword, as trafficking deficiencies in one gene can have multigenic dominant-negative effects. As the two somatic *KCNA2* alleles are presumably under the same transcriptional control, our experiments suggest that that the neurons of the heterozygote patient will have ∼20% K_V_1.2 conductance (Fig. 3 *A* and *B*). Effects on other subunits will depend on their relative expression levels. We posit that epileptogenesis is mainly driven by K_V_1.2 loss of function, with some K_V_1.4 decrease in neurons where these genes are coexpressed. K_V_1.1 are also likely suppressed by K_V_1.2(F233S), as they traffic less proficiently than K_V_1.2 or K_V_1.4 due to their potent ER retention signal (35). Loss of K_V_1.2 function should prolong action potentials in both excitatory and inhibitory neurons. In the former, this can cause increased synaptic release (4). On the other hand, fast-gating K_V_3 channels are more abundant in inhibitory neurons (57–59), where they delimit action potential duration (60, 61); thus, some inhibitory synaptic release is protected from loss of K_V_1.2. The resulting imbalance of excitatory and inhibitory signals could veer neuronal circuits toward epilepsy (62).

## Methods

This study was reviewed and deemed exempt by the Children’s Hospital Los Angeles Institutional Review Board. A consent form by Children's Hospital Los Angeles compliant with the Health Insurance Portability and Accountability Act of 1996 (HIPAA) was signed by the patient's parents. DNA was extracted from the peripheral blood of the patient and each of his unaffected parents as comparators using a commercially available kit (Promega Maxwell RSC DNA Extraction Kit). Original trafficking-assay and K_V_1.4 plasmids were described in (27) and (63), respectively. Confocal microscopy, cut-open oocyte Vaseline gap (64), and VCF (65) experiments were performed as previously described (8). For some flow cytometry experiments, the methodology [previously described in Pantazis et al. (8)] was expanded to four colors to evaluate synthesis and surface trafficking of two constructs simultaneously. Two-electrode voltage-clamp (TEVC) experiments were performed in an OpusXpress 6000A (Axon Instruments) parallel voltage clamp to facilitate testing of a large number of cells.

## Supplementary Material

Supplementary File

## Data Availability

All study data are included in the article and/or *SI Appendix*.

## Change History

May 12, 2022: Due to a prepress vendor’s error, the Abstract has been updated to correct a typographical error.

## References

[1] W. Stühmer , Molecular basis of functional diversity of voltage-gated potassium channels in mammalian brain. EMBO J. 8, 3235–3244 (1989). 2555158 10.1002/j.1460-2075.1989.tb08483.x PMC401447

[2] J. S. Trimmer, Subcellular localization of K^+^ channels in mammalian brain neurons: Remarkable precision in the midst of extraordinary complexity. Neuron 85, 238–256 (2015). 25611506 10.1016/j.neuron.2014.12.042 PMC4303806

[3] B. P. Bean, The action potential in mammalian central neurons. Nat. Rev. Neurosci. 8, 451–465 (2007). 17514198 10.1038/nrn2148

[4] M. H. Kole, J. J. Letzkus, G. J. Stuart, Axon initial segment K_v_1 channels control axonal action potential waveform and synaptic efficacy. Neuron 55, 633–647 (2007). 17698015 10.1016/j.neuron.2007.07.031

[5] M. Sheng, Y. J. Liao, Y. N. Jan, L. Y. Jan, Presynaptic A-current based on heteromultimeric K^+^ channels detected in vivo. Nature 365, 72–75 (1993). 8361540 10.1038/365072a0

[6] S. K. Coleman, J. Newcombe, J. Pryke, J. O. Dolly, Subunit composition of K_v_1 channels in human CNS. J. Neurochem. 73, 849–858 (1999). 10428084 10.1046/j.1471-4159.1999.0730849.x

[7] S. Masnada , Clinical spectrum and genotype-phenotype associations of KCNA2-related encephalopathies. Brain 140, 2337–2354 (2017). 29050392 10.1093/brain/awx184

[8] A. Pantazis , Tracking the motion of the K_V_1.2 voltage sensor reveals the molecular perturbations caused by a de novo mutation in a case of epilepsy. J. Physiol. 598, 5245–5269 (2020). 32833227 10.1113/JP280438 PMC8923147

[9] J. H. Döring , Refining genotypes and phenotypes in *KCNA2*-related neurological disorders. Int. J. Mol. Sci. 22, 2824 (2021). 33802230 10.3390/ijms22062824 PMC7999221

[10] P. Imbrici ., A Novel *KCNA2* Variant in a Patient with Non-Progressive Congenital Ataxia and Epilepsy: Functional Characterization and Sensitivity to 4-Aminopyridine. Int. J. Mol. Sci. 22, 9913 (2021). 34576077 10.3390/ijms22189913 PMC8469797

[11] X. Tao, A. Lee, W. Limapichat, D. A. Dougherty, R. MacKinnon, A gating charge transfer center in voltage sensors. Science 328, 67–73 (2010). 20360102 10.1126/science.1185954 PMC2869078

[12] I. G. Ishida, G. E. Rangel-Yescas, J. Carrasco-Zanini, L. D. Islas, Voltage-dependent gating and gating charge measurements in the K_v_1.2 potassium channel. J. Gen. Physiol. 145, 345–358 (2015). 25779871 10.1085/jgp.201411300 PMC4380214

[13] J. J. Lacroix, F. Bezanilla, Control of a final gating charge transition by a hydrophobic residue in the S2 segment of a K^+^ channel voltage sensor. Proc. Natl. Acad. Sci. U.S.A. 108, 6444–6449 (2011). 21464282 10.1073/pnas.1103397108 PMC3081032

[14] T. Kalstrup, R. Blunck, S4-S5 linker movement during activation and inactivation in voltage-gated K^+^ channels. Proc. Natl. Acad. Sci. U.S.A. 115, E6751–E6759 (2018). 29959207 10.1073/pnas.1719105115 PMC6055142

[15] F. Tombola, M. M. Pathak, E. Y. Isacoff, How does voltage open an ion channel? Annu. Rev. Cell Dev. Biol. 22, 23–52 (2006). 16704338 10.1146/annurev.cellbio.21.020404.145837

[16] S. I. Börjesson, F. Elinder, Structure, function, and modification of the voltage sensor in voltage-gated ion channels. Cell Biochem. Biophys. 52, 149–174 (2008). 18989792 10.1007/s12013-008-9032-5

[17] B. Chanda, F. Bezanilla, A common pathway for charge transport through voltage-sensing domains. Neuron 57, 345–351 (2008). 18255028 10.1016/j.neuron.2008.01.015

[18] R. Blunck, Z. Batulan, Mechanism of electromechanical coupling in voltage-gated potassium channels. Front. Pharmacol. 3, 166 (2012). 22988442 10.3389/fphar.2012.00166 PMC3439648

[19] M. O. Jensen , Mechanism of voltage gating in potassium channels. Science 336, 229–233 (2012). 22499946 10.1126/science.1216533

[20] C. S. Schwaiger, S. I. Liin, F. Elinder, E. Lindahl, The conserved phenylalanine in the K^+^ channel voltage-sensor domain creates a barrier with unidirectional effects. Biophys. J. 104, 75–84 (2013). 23332060 10.1016/j.bpj.2012.11.3827 PMC3540256

[21] S. B. Long, X. Tao, E. B. Campbell, R. MacKinnon, Atomic structure of a voltage-dependent K^+^ channel in a lipid membrane-like environment. Nature 450, 376–382 (2007). 18004376 10.1038/nature06265

[22] M. Lek ; Exome Aggregation Consortium, Analysis of protein-coding genetic variation in 60,706 humans. Nature 536, 285–291 (2016). 27535533 10.1038/nature19057 PMC5018207

[23] M. J. Landrum , ClinVar: Improving access to variant interpretations and supporting evidence. Nucleic Acids Res. 46, D1062–D1067 (2018). 29165669 10.1093/nar/gkx1153 PMC5753237

[24] P. D. Stenson , The Human Gene Mutation Database: Towards a comprehensive repository of inherited mutation data for medical research, genetic diagnosis and next-generation sequencing studies. Hum. Genet. 136, 665–677 (2017). 28349240 10.1007/s00439-017-1779-6 PMC5429360

[25] N. L. Sim , SIFT web server: Predicting effects of amino acid substitutions on proteins. Nucleic Acids Res. 40, W452–W457 (2012). 22689647 10.1093/nar/gks539 PMC3394338

[26] J. M. Schwarz, D. N. Cooper, M. Schuelke, D. Seelow, MutationTaster2: Mutation prediction for the deep-sequencing age. Nat. Methods 11, 361–362 (2014). 24681721 10.1038/nmeth.2890

[27] C. Gu, Y. N. Jan, L. Y. Jan, A conserved domain in axonal targeting of K_v_1 (Shaker) voltage-gated potassium channels. Science 301, 646–649 (2003). 12893943 10.1126/science.1086998

[28] J. Lu, J. M. Robinson, D. Edwards, C. Deutsch, T1-T1 interactions occur in ER membranes while nascent Kv peptides are still attached to ribosomes. Biochemistry 40, 10934–10946 (2001). 11551188 10.1021/bi010763e

[29] E. R. Liman, J. Tytgat, P. Hess, Subunit stoichiometry of a mammalian K^+^ channel determined by construction of multimeric cDNAs. Neuron 9, 861–871 (1992). 1419000 10.1016/0896-6273(92)90239-a

[30] S. Akhtar, O. Shamotienko, M. Papakosta, F. Ali, J. O. Dolly, Characteristics of brain K_v_1 channels tailored to mimic native counterparts by tandem linkage of alpha subunits: Implications for K^+^ channelopathies. J. Biol. Chem. 277, 16376–16382 (2002). 11859070 10.1074/jbc.M109698200

[31] E. Y. Isacoff, Y. N. Jan, L. Y. Jan, Evidence for the formation of heteromultimeric potassium channels in Xenopus oocytes. Nature 345, 530–534 (1990). 2112229 10.1038/345530a0

[32] J. P. Ruppersberg , Heteromultimeric channels formed by rat brain potassium-channel proteins. Nature 345, 535–537 (1990). 2348860 10.1038/345535a0

[33] H. Misonou, J. S. Trimmer, Determinants of voltage-gated potassium channel surface expression and localization in Mammalian neurons. Crit. Rev. Biochem. Mol. Biol. 39, 125–145 (2004). 15596548 10.1080/10409230490475417

[34] D. Li, K. Takimoto, E. S. Levitan, Surface expression of K_v_1 channels is governed by a C-terminal motif. J. Biol. Chem. 275, 11597–11602 (2000). 10766775 10.1074/jbc.275.16.11597

[35] L. N. Manganas , Identification of a trafficking determinant localized to the K_v_1 potassium channel pore. Proc. Natl. Acad. Sci. U.S.A. 98, 14055–14059 (2001). 11698661 10.1073/pnas.241403898 PMC61166

[36] L. N. Manganas, J. S. Trimmer, Subunit composition determines K_v_1 potassium channel surface expression. J. Biol. Chem. 275, 29685–29693 (2000). 10896669 10.1074/jbc.M005010200

[37] J. Tseng-Crank, J. A. Yao, M. F. Berman, G. N. Tseng, Functional role of the NH2-terminal cytoplasmic domain of a mammalian A-type K channel. J. Gen. Physiol. 102, 1057–1083 (1993). 7907648 10.1085/jgp.102.6.1057 PMC2229192

[38] Si. Kondoh, K. Ishii, Y. Nakamura, N. Taira, A mammalian transient type K^+^ channel, rat K_v_1.4, has two potential domains that could produce rapid inactivation. J. Biol. Chem. 272, 19333–19338 (1997). 9235930 10.1074/jbc.272.31.19333

[39] L. M. Mannuzzu, M. M. Moronne, E. Y. Isacoff, Direct physical measure of conformational rearrangement underlying potassium channel gating. Science 271, 213–216 (1996). 8539623 10.1126/science.271.5246.213

[40] M. Priest, F. Bezanilla, Functional site-directed fluorometry. Adv. Exp. Med. Biol. 869, 55–76 (2015). 26381940 10.1007/978-1-4939-2845-3_4

[41] A. J. Horne, C. J. Peters, T. W. Claydon, D. Fedida, Fast and slow voltage sensor rearrangements during activation gating in K_v_1.2 channels detected using tetramethylrhodamine fluorescence. J. Gen. Physiol. 136, 83–99 (2010). 20584892 10.1085/jgp.201010413 PMC2894543

[42] S. D. Demo, G. Yellen, The inactivation gate of the Shaker K^+^ channel behaves like an open-channel blocker. Neuron 7, 743–753 (1991). 1742023 10.1016/0896-6273(91)90277-7

[43] J. P. Ruppersberg, R. Frank, O. Pongs, M. Stocker, Cloned neuronal IK(A) channels reopen during recovery from inactivation. Nature 353, 657–660 (1991). 1922383 10.1038/353657a0

[44] C. M. Armstrong, F. Bezanilla, Inactivation of the sodium channel. II. Gating current experiments. J. Gen. Physiol. 70, 567–590 (1977). 591912 10.1085/jgp.70.5.567 PMC2228472

[45] M. J. Roux, R. Olcese, L. Toro, F. Bezanilla, E. Stefani, Fast inactivation in Shaker K^+^ channels. Properties of ionic and gating currents. J. Gen. Physiol. 111, 625–638 (1998). 9565401 10.1085/jgp.111.5.625 PMC2217138

[46] N. Savalli, A. Kondratiev, S. B. de Quintana, L. Toro, R. Olcese, Modes of operation of the BKCa channel beta2 subunit. J. Gen. Physiol. 130, 117–131 (2007). 17591990 10.1085/jgp.200709803 PMC2154362

[47] J. Xu, W. Yu, Y. N. Jan, L. Y. Jan, M. Li, Assembly of voltage-gated potassium channels. Conserved hydrophilic motifs determine subfamily-specific interactions between the alpha-subunits. J. Biol. Chem. 270, 24761–24768 (1995). 7559593 10.1074/jbc.270.42.24761

[48] D. G. Gagnon, F. Bezanilla, The contribution of individual subunits to the coupling of the voltage sensor to pore opening in Shaker K channels: Effect of ILT mutations in heterotetramers. J. Gen. Physiol. 136, 555–568 (2010). 20974773 10.1085/jgp.201010487 PMC2964516

[49] J. L. Ledwell, R. W. Aldrich, Mutations in the S4 region isolate the final voltage-dependent cooperative step in potassium channel activation. J. Gen. Physiol. 113, 389–414 (1999). 10051516 10.1085/jgp.113.3.389 PMC2222902

[50] C. Deutsch, Potassium channel ontogeny. Annu. Rev. Physiol. 64, 19–46 (2002). 11826262 10.1146/annurev.physiol.64.081501.155934

[51] L. Tu, J. Wang, A. Helm, W. R. Skach, C. Deutsch, Transmembrane biogenesis of K_v_1.3. Biochemistry 39, 824–836 (2000). 10651649 10.1021/bi991740r

[52] L. Tu, C. Deutsch, Determinants of helix formation for a K_v_1.3 transmembrane segment inside the ribosome exit tunnel. J. Mol. Biol. 429, 1722–1732 (2017). 28478285 10.1016/j.jmb.2017.04.022 PMC5511032

[53] S. K. Tiwari-Woodruff, C. T. Schulteis, A. F. Mock, D. M. Papazian, Electrostatic interactions between transmembrane segments mediate folding of Shaker K^+^ channel subunits. Biophys. J. 72, 1489–1500 (1997). 9083655 10.1016/S0006-3495(97)78797-6 PMC1184345

[54] J. Zhu , The K_v_1.2 potassium channel: The position of an N-glycan on the extracellular linkers affects its protein expression and function. Brain Res. 1251, 16–29 (2009). 19056359 10.1016/j.brainres.2008.11.033

[55] D. A. Thayer, S. B. Yang, Y. N. Jan, L. Y. Jan, N-linked glycosylation of K_v_1.2 voltage-gated potassium channel facilitates cell surface expression and enhances the stability of internalized channels. J. Physiol. 594, 6701–6713 (2016). 27377235 10.1113/JP272394 PMC5108895

[56] F. H. Yu, W. A. Catterall, The VGL-chanome: A protein superfamily specialized for electrical signaling and ionic homeostasis. Sci. STKE 2004, re15 (2004). 15467096 10.1126/stke.2532004re15

[57] C. Sekirnjak , Subcellular localization of the K^+^ channel subunit K_v_3.1b in selected rat CNS neurons. Brain Res. 766, 173–187 (1997). 9359601 10.1016/s0006-8993(97)00527-1

[58] A. Erisir, D. Lau, B. Rudy, C. S. Leonard, Function of specific K(+) channels in sustained high-frequency firing of fast-spiking neocortical interneurons. J. Neurophysiol. 82, 2476–2489 (1999). 10561420 10.1152/jn.1999.82.5.2476

[59] B. Tasic , Adult mouse cortical cell taxonomy revealed by single cell transcriptomics. Nat. Neurosci. 19, 335–346 (2016). 26727548 10.1038/nn.4216 PMC4985242

[60] M. J. Rowan, E. Tranquil, J. M. Christie, Distinct Kv channel subtypes contribute to differences in spike signaling properties in the axon initial segment and presynaptic boutons of cerebellar interneurons. J. Neurosci. 34, 6611–6623 (2014). 24806686 10.1523/JNEUROSCI.4208-13.2014 PMC4012316

[61] A. J. Labro, M. F. Priest, J. J. Lacroix, D. J. Snyders, F. Bezanilla, K_v_3.1 uses a timely resurgent K(+) current to secure action potential repolarization. Nat. Commun. 6, 10173 (2015). 26673941 10.1038/ncomms10173 PMC4703866

[62] D. A. McCormick, D. Contreras, On the cellular and network bases of epileptic seizures. Annu. Rev. Physiol. 63, 815–846 (2001). 11181977 10.1146/annurev.physiol.63.1.815

[63] K. Nakahira, G. Shi, K. J. Rhodes, J. S. Trimmer, Selective interaction of voltage-gated K+ channel beta-subunits with alpha-subunits. J. Biol. Chem. 271, 7084–7089 (1996). 8636142 10.1074/jbc.271.12.7084

[64] A. Pantazis, R. Olcese, “Cut-open oocyte voltage-clamp technique” in Encyclopedia of Biophysics, G. Roberts, A. Watts, European Biophysical Societies, Eds. (Springer, Berlin, Heidelberg, 2019), pp. 1–9.

[65] C. S. Gandhi, R. Olcese, The voltage-clamp fluorometry technique. Methods Mol. Biol. 491, 213–231 (2008). 18998096 10.1007/978-1-59745-526-8_17

