# Supplementary material for: An epilepsy-associated KV1.2 charge-transfer-center mutation impairs KV1.2 and KV1.4 trafficking
Source: Proc Natl Acad Sci U S A. 2022 Apr 19;119(17):e2113675119. doi: 10.1073/pnas.2113675119 (PMC9169947; doi:10.1073/pnas.2113675119)
Supplement: Supplementary File [file pnas.2113675119.sapp.pdf]

PNAS
www.pnas.org

Supplementary Information for

An epilepsy-associated $K_V1.2$ charge-transfer-center mutation suppresses $K_V1.2$ and $K_V1.4$ subunit trafficking

Michelle Nilsson, Sarah H. Lindström, Maki Kaneko, Kaiqian Wang, Teresa Mínguez-Viñas, Marina Angelini, Federica Steccanella, Deborah Holder, Michela Ottolia, Riccardo Olcese, Antonios Pantazis

Corresponding author: Antonios Pantazis
Email: antonios.pantazis@liu.se

This PDF file includes:

Supplementary text
Figures S1 to S6
Table S1
SI References

Supplementary Information Text

Tetramerization Model of specific stoichiometric trafficking: Alternatives and Assumptions

In this study, we propose that 3^{WT}:1^{F233S} $K_V1.2$ hetero-tetramers are trafficking-capable, whereas all other stoichiometries are not. We also propose that only 3:1 and 2:2 $K_V1.4$:$K_V1.2$(F233S) hetero-tetramers can traffic. These findings primarily arise from the combination of "applied transcriptomics" electrophysiology (stringently controlling the RNA level of specific subunits over a broad range, at the single-cell level) with a model of binomial subunit tetramerization (Figs.5A & 7A); but they are also supported by results from additional experiments: concatemer electrophysiology, flow cytometry / immunocytochemistry and voltage-clamp fluorometry.

First, one might ask: "why hypothesize that specific stoichiometries are differentially trafficking-capable?" An alternative possibility is that all F233S-containing stoichiometries are able to traffic equally, but are less capable to do so than wild-type $K_V1.2$ or $K_V1.4$ homo-tetramers. In the case of $K_V1.2$, while we cannot directly observe 3^{WT}:1^{F233S} heteromers, their preferential trafficking over 2^{WT}:2^{F233S} channels is supported by (i) the very poor trafficking of $K_V1.2$(WT-F233S) concatemers, which emulate the 2:2 stoichiometry (Fig.5B,C) and (ii) the clear fluorescence signals from rescued F233S subunits in flow cytometry and VCF experiments (Figs. 4, S1, 8B). If all heteromers trafficked with 5% efficiency, it would be very difficult to detect them by optical methods. We simulated this condition with the binomial subunit tetramerization model by allowing all heteromers to traffic equally with specified efficiencies (Fig.S5): none of these models described the data as well. In the case of $K_V1.4$, the answer is more clear: the $K_V1.4$-$K_V1.2$(F233S) concatemer, which emulates the 2:2 stoichiometry, trafficked with 100% efficiency (Fig.7D,E). If all stoichiometries of $K_V1.4$/$K_V1.2$(F233S) could traffic equally efficiently, then we would expect the purple curve to account for the data in Fig.7A. Ultimately, we cannot exclude some degree of trafficking of "non-permissible" stoichiometries; in fact, $K_V1.2$(WT-F233S) concatemers do traffic with very low efficiency (5-6%). Our view is that the efficiency of heteromeric trafficking is *primarily* determined by the stoichiometric composition.

We must also consider two assumptions of the binomial subunit tetramerization models: (a) the total macroscopic conductance (G_{total}) reflects the number of channels per condition, (b) $K_V1.2$(F233S) subunits assemble in an unbiased manner with wild-type $K_V1.2$ and $K_V1.4$.

a) The total macroscopic conductance (G_{total}) reflects the number of channels per condition.
The model outputs the frequency of specific tetrameric stoichiometries; these are compared to macroscopic conductance data, which are assumed to reflect the number of channels in the membrane. While the number of channels does contribute to the macroscopic conductance, two additional factors are maximal open probability (max P_O) and single-channel conductance (γ). If max P_O and γ were the same in all stoichiometries, then G_{total} alone would be an accurate measure of number of channels for all tested conditions.
If $K_V1.2$(F233S) subunit inclusion decreased max P_O and γ, instead of channel trafficking, then one would expect no $K_V1.2$(WT) or $K_V1.4$ subunit sequestration; however, the latter is clearly evidenced in immunocytochemical experiments (Figs. 4, 6, S1).

If $K_V1.2$ co-assembly with $K_V1.4$ resulted in heteromers with higher max P_O or γ than $K_V1.4$ homomers, it might be possible that the data in Fig.7A could be accounted for by only 3:1 $K_V1.4$:$K_V1.2$(F233S) hetero-tetramers, excluding the 2:2 stoichiometry. This possibility is unlikely since the $K_V1.4$-$K_V1.2$(F233S) concatemer supports 2:2 subunit trafficking (Fig.7D,E).

Ultimately, we cannot altogether exclude that some combination of max P_O, γ and trafficking effects gave rise to the experimental data. The occurrence of such combinations is less parsimonious than simple trafficking defects; and the model findings in general are very well supported by experimental results.

b) $K_V1.2$(F233S) subunits assemble in an unbiased manner with wild-type $K_V1.2$ and $K_V1.4$.

The binomial distribution of heteromers arises from the unbiased association of wild-type and mutant subunits. This is a reasonable assumption as (i) all $K_V1.2$ and $K_V1.4$ subunits have an intact N-terminal tetramerization domain that facilitates association even as the nascent peptide is

assembled by the ribosome (1); (ii) the mixed-subunit RNA is introduced to the oocyte at precisely the same place and time.

We can challenge this idea by introducing a negative association bias; *i.e.*, wild-type K_V1.2 subunits are less likely to co-assemble with K_V1.2(F233S). Effectively, this means that the availability of F233S subunits is diminished, which can be calculated by reducing the molar proportion of F233S by a factor <1. Conversely, multiplying by a factor >1 increases its effective concentration. We explore both ideas in Fig.S6. Decreasing F233S effective concentration narrows the spread of the curves, such that K_V1.2 data are now better described by the black model (no heteromers / F233S-subunit rescue; Fig.S6B); and the K_V1.4 data by the green model (no 2:2 stoichiometry; Fig.S6E). However, both models are contradicted by experimental results from flow cytometry, VCF and concatemer experiments. Increasing F233S effective concentration splays out the curves. For K_V1.2, this means that data are best described by the cyan model (2:2 stoichiometry allowed; Fig.S6C); however this is inconsistent with concatemer experiments. No condition with bias >1 could explain the K_V1.4 data better than unbiased association (Fig.S6F). To summarize, the “applied transcriptomics data”, in combination with other experiments in this work, favor unbiased heteromeric channel assembly.

As discussed, the data go a long way in rejecting most alternatives to specific stoichiometry trafficking. We cannot completely exclude contributions from other mechanisms (such as non-stringent stoichiometric trafficking, open probability / single-channel conductance, biased subunit association), nor is this necessary: the main conclusions of the model are both falsifiable and well supported by our experimental data, all the more impressive as the model carries no free parameters: model predictions are simply overlaid, not fit to the data.

Materials & Methods

Patient genetic testing

A HIPAA-compliant consent form by Children’s Hospital Los Angeles was signed by the patient’s parents. DNA was extracted from the peripheral blood of the patient and each of his unaffected parents as comparators using a commercially available kit (Promega Maxwell RSC DNA Extraction Kit). The exome sequencing library was generated for proband DNA using the Agilent SureSelect Human All Exon V6+Mito kit. Captured DNA fragments were then sequenced using the Illumina Nextseq 500 sequencing system, with 2$\times$100 basepair (bp) paired-end reads. Single nucleotide variants (SNVs) and small insertions and deletions (<10 bp) were detected by mapping and comparing the DNA sequences with the human reference genome (GRCh37-hg19). Of all the variants identified by exome sequencing, a list of rare variants (minor allele frequency <1%) located within a predefined set of 224 epilepsy associated genes (Table S1) was generated. These variants were further annotated and analyzed using Alissa Interpret (Agilent). The genes included in this panel are known to be associated with primary epilepsy, or associated with syndromes in which epilepsy is a commonly observed feature. The genes (Table S1) were evaluated for single nucleotide substitutions and small indels. Both parents and the patient were tested for the variant by Sanger sequencing (Fig.1D). Variant interpretation was done according to the standards and guidelines of variant interpretation by American College of Medical Genetics and Genomics and Association for Molecular Pathology (2).

Molecular biology

Site-directed mutagenesis (SDM) to introduce F233S, A291C, *etc.*, was performed with a high-fidelity *Pfu* polymerase (Agilent 600850), unless otherwise stated. All molecular biology reagents were provided from New England Biolabs, and all synthetic oligonucleotides from Integrated DNA Technologies, unless stated otherwise. All molecular biology operations were confirmed by sequencing.

Immunocytochemistry constructs

For initial two-color immunocytochemistry studies (Fig.2B-D), a plasmid containing rat K_V1.2 with an N-terminally fused EGFP tag (in the pEGFP-C1 vector) and an extracellular HA site (between transmembrane helices S1 and S2) was used; it was a generous gift from Lily Y. Jan (3, 4).

To incorporate a bungarotoxin binding site (BBS; TGG CGG TAC TAC GAG AGC AGC CTG GAG CCC TAC CCC GAC) in K_V1.2(HA) and K_V1.2(HA,F233S) constructs, the HA tag was excised using *BstEII* and *EcoRI* and replaced by a synthetic BBS-containing oligo. The resulting constructs had a BBS at the same position as the excised HA tag.

Rat brain K_V1.4 in the GW1 plasmid was gifted by James S. Trimmer (5). A hemagglutinin (HA) tag (TAT CCG TAC GAC GTC CCA GAC TAT GCG) was introduced at the S1-S2 extracellular linker after position G346. This was performed by excising a portion of the K_V1.4 N-terminus between unique sites *NotI* and *BsmI*, and replacing it with a gBlocks synthetic fragment including the HA tag sequence. The new construct was evaluated by cut-open oocyte Vaseline gap electrophysiology (see below): it exhibited fast N-type inactivation, and activated with a voltage-dependence similar to the tag-less channel $\Delta V_{0.5}$=−13±4mV (*n*=4 per condition).

For four-color immunocytochemistry (Figs.4, 6, S1), HA-tagged channels were subcloned into pCDNA3-EGFP vectors (Addgene #13031, http://n2t.net/addgene:13031 ; RRID:Addgene_13031, a kind gift from Doug Golenbock) to make HA-tagged channels with an N-terminally-fused EGFP. Similarly, BBS-tagged channels were subcloned into pCDNA3-mRFP vectors (Addgene #13032, http://n2t.net/addgene:13032 ; RRID:Addgene_13032, a kind gift from Doug Golenbock).

Electrophysiology constructs

The rat K_V1.2 cDNA construct in pMAX oocyte expression vector was a kind gift from Benoît Roux.

Rat K_V1.4 cDNA was subcloned from GW1 to pMAX. The inactivation-removed (IR, Δ2-61) construct (6) was generated by excluding 177 bp immediately following the K_V1.4 ATG using the Q5 Site-Directed Mutagenesis kit.

To construct concatemeric dimers, a *BamHI* site was first introduced upstream of K_V1.4, to match the 5′ untranslated region (UTR) of K_V1.2, by SDM. The existing K_V1.*x* (*x*=2, 2(F233S) or 4) open reading frames (ORFs) would then become the C-terminal domains of the concatemer. N-terminal K_V1.*x* domains, originally generated by PCR amplification, were then introduced, along with a synthetic linker sequence modified from the 5′ UTR of the *Xenopus* β-globin gene (7, 8). The N-terminal ORFs lacked a stop codon, and the sequence between the two K_V1.*x* domains was: GCT AGC GAT ACG AAG GAG CGA GGA AAC CTC TTC ACG TCA ACC GGA TCC **GCC GCC ACC ATT** (*NheI* and *BamHI* sites are underlined at the 5′ and 3′, respectively; bold indicates preserved pMAX sequence preceding the start codon of the C-terminal K_V1.*x* domain), which corresponds to the 20-residue linker: ASDTKERGNLFTSTGSAATI.

pMAX plasmids were linearized using *PacI*, transcribed to cRNA *in vitro* (T7 mMESSAGE MACHINE, Thermo Fisher Scientific) and stored at −80 °C in RNA storage solution (Thermo Fisher Scientific). cRNA was quantified spectrophotometrically and evaluated by gel electrophoresis.

Immunocytochemistry & flow cytometry

COS-7 cell culture and transfection: COS-7 cells (ECACC 87021302) were grown in Complete Culture Medium containing: Dulbecco's modified Eagle's medium (DMEM)/F-12 Nutrient Mixture (1:1) (Gibco), heat-inactivated fetal bovine serum (FBS) (10%), penicillin (100 units), streptomycin (100 mg/ml) and glutamine (0.5 mM). Cultures were incubated at 37°C with 5% CO_2 and passaged twice per week up to p-24. Cells used for flow-cytometry (FC) were seeded in 12-well plates (approx. 30,000 cells/well). Cells used for immunocytochemistry (ICC) were seeded in 35

mm glass-bottom dishes (approx. 15,000 cells/dish). Cells were transiently transfected 24 hr after seeding using TransIT-X2 Dynamic Delivery System (Mirus; pEGFP-C1 constructs, Fig.2B-D; ICC: 1.2µl TransIT-X2 : 0.6µg DNA; FC: 4µl TransIT-X2 : 1µg DNA) or jetOPTIMUS (Polyplus transfection; pCDNA3-EGFP/mRFP1 constructs, Figs.4, 6, S1; 1µl jetOPTIMUS : 1µg DNA).

Immunocytochemistry / confocal microscopy: Primary (Rat anti-HA; Roche 3F10) and secondary (AlexaFluor 568 conjugated goat anti-rat IgG(H+L); Invitrogen A-11077) antibodies were diluted 1:200 and 1:1000, respectively, in blocking solution. 48 hr post-transfection, COS-7 cells were rinsed with ice-chilled phosphate-buffered saline (PBS) supplemented with 0.9 mM Ca^{2+} and 0.5 mM Mg^{2+} (Gibco 14040-133). Cells were fixed with 4% paraformaldehyde in PBS (without Ca^{2+} or Mg^{2+}, 5 min), and washed with PBS (once quickly, then 3×5 min each) prior to incubation with blocking solution (2 ml of 5% normal goat serum in PBS) for at least 1 hr at room temperature. Blocking solution was replaced with primary antibody solution (0.5 ml) and cells were incubated at room temperature for at least 2 hrs. Cells were washed with PBS prior to incubation with secondary antibody solution (0.5 ml, 1 hr at room temperature). Unbound antibodies were washed off with PBS and a coverslip was mounted over the glass-bottom of each dish using ProLong Glass Antifade Mountant (Invitrogen P36982). Mountant was allowed to set for 18-24 hr at room temperature, then samples stored at 4°C. Confocal images were acquired using a Zeiss LSM 800 Microscope with Zen 2.3 software. A z-stack was acquired for each cell (z-section interval = 0.27 µm). Zen Analysis software was used to create an orthogonal projection of 2 sequential z-sections. Final images were prepared for publication using Adobe Photoshop CS6. Identical adjustments to levels, sharpness and pixel sampling were performed on all samples.

Flow cytometry: To quantify K_V1.2 subunit cell-surface expression (Fig.2D), COS-7 cells were harvested by trypsinization (0.05%) 48 hr post-transfection. Cells were washed with Dulbecco's PBS (DPBS, Gibco) and pelleted at 400 *g* for 5 min at 4°C. Cells were stained with Zombie Violet viability dye (BioLegend) and anti-HA conjugated Alexa Fluor 647 (Alexa Fluor 647 anti-HA.11 Epitope Tag Antibody; BioLegend Cat# 682404, RRID:AB_2566616) as previously described (4, 9). Cells were detected using a Gallios Flow Cytometer (Beckman Coulter Life Sciences) as previously described (4). The fraction of anti-HA positive cells in the EGFP positive population, normalized to wild type K_V1.2(HA) measurements was calculated.

To evaluate K_V1.2(F233S) subunit surface trafficking in the presence or absence of K_V1.2(WT) or K_V1.4 subunits and *vice versa* (Figs.4, 6, S1), cells were stained with 8 µg/ml anti-HA conjugated Brilliant Violet 421 (Brilliant Violet 421 anti-HA.11 Epitope Tag Antibody; BioLegend Cat# 682405, RRID: AB_2716037) and 1 µg/ml α-bungarotoxin-conjugated Alexa Fluor 647 (Invitrogen, #B35450) in 100 µl DPBS/5% FBS for 45 min at 4°C. Cells were washed with (i) DPBS/5% FBS and (ii) DPBS. Finally, a single-cell suspension was prepared using pre-separation filters (Miltenyi Biotec) (70µm) and stored at 4°C in the dark until detection later the same day (4, 9). Cells were detected using a BD FACSAria III Cell Sorter. Brilliant Violet 421, EGFP, mRFP1 and BTX 647 were excited at 405, 488, 561 and 633 nm respectively. Detection was filtered for wavelengths 450/40, 550/30, 610/20 and 660/20 nm. Cell clumps or fragments were excluded using forward and side scatter gating. Positive gates for Brilliant Violet 421, EGFP, mRFP1 and BTX 647 were set using untransfected cells, negative controls for anti-HA or BTX and fluorescence-minus-one controls. Spillover between channels were compensated in single color controls: EGFP-K_V1.2(WT) or mRFP1-K_V1.2(WT). Only EGFP and mRFP1 double-positive cells per replicate were counted. Data were analyzed using Kaluza Analysis 1.3 (Beckman Coulter Life Sciences) and Microsoft Excel.

Electrophysiology & voltage-clamp fluorometry

Oocyte preparation: All animal experiments were approved by the Linköping University Animal Care and Use Committee. Defolliculated *Xenopus laevis* oocytes were either purchased from Ecocyte or isolated and prepared from locally-kept *Xenopus laevis* (Nasco, WI, USA) as follows: Frogs were anesthetized with 1.4g/L tricaine (ethyl 3-aminobenzoate methanesulfonate, Sigma #A5040). Lobes of ovaries were removed through an abdominal incision and placed into Ca^{2+}-free OR-2 solution (in mM: 82.5 NaCl, 2.5 KCl, 1 $MgCl_2$ and 5 HEPES; pH adjusted to 7.4 by

NaOH) for further processing. Frogs were treated with analgesics (5mg/mL Marcain; Astra Zeneca & 2% Xylocain gel; Aspen) and the incision was sutured, prior to being returned to a recovery aquarium for post-surgical monitoring. Ovaries were cut into small clusters of 5-7 oocytes, then enzymatically treated with Liberase TM (7 Units/batch; Roche 05401127001) in ~10 ml of OR-2 with agitation using an orbital shaker for 25-40 min. Liberase was removed by washing with OR-2 solution, then manual agitation for 30-60 min was employed to remove follicular layers. Mature (stage V-VI) defolliculated oocytes were selected and stored at 17°C in SOS (in mM: 100 NaCl, 2 KCl, 1.8 $CaCl_2$, 1 $MgCl_2$ and 5 HEPES; pH adjusted to 7.0 by NaOH).

Parallel two-electrode voltage clamp (TEVC) recordings: Oocytes were injected with 50 nl of cRNA solution using a nanoinjector (UMP3T-1, World Precision Instruments). Specific cRNA amounts used are mentioned in their corresponding experiments. One day after injection, oocytes were recorded using a medium throughput robot capable of eight simultaneous TEVC recordings (OpusXpress 6000A Parallel Oocyte Voltage Clamp; Axon Instruments). Recording electrodes were pulled from TW150F-6 thin-wall capillary glass (World Precision Instruments) on a P-1000 horizontal puller (Sutter Instruments). Only electrodes with resistance ≤1 Ω (I-electrodes), or ≤1.5 Ω (V-electrodes) were used. Electrodes were filled with 3 M KCl. The oocyte recording chambers were perfused continuously with SOS. Once oocytes in all chambers were correctly impaled by both electrodes, oocytes were clamped at a holding potential of –80mV. P/–6 subtraction was used to limit capacitative transients. For K_V1.2(WT/F233S) experiments (Figs. 3, 5A, S2A,B), voltage-dependent activation was evaluated using a series of 100ms test-pulses from –80mV to 120mV in 10-mV increments. For experiments including K_V1.4 subunits (Figs. 7A-C, S3), voltage-dependent activation was evaluated using a series of 100ms test-pulses (from –90mV to 100mV in 10-mV increments). A pre-pulse to –120mV for 800ms preceded each test-pulse to completely relieve N-type inactivation. A 10 s inter-pulse interval was used in all recordings. Current was low-pass filtered to 2 kHz and acquired at 6.25 kHz using the OpusXpress digitizer and OpusXpress 1.1 software (Axon Instruments).

Cut-open oocyte Vaseline gap (COVG) recordings: COVG is a low-noise, fast voltage clamp technique (10-12). A Clampator One CA-1 amplifier (Dagan) was used in COVG mode. Its C95 capacitor was replaced by one of 470 pF to improve capacitative transient compensation. The oocyte was placed in a triple-compartment Perspex chamber, with a diameter of 600 µm for the top and bottom apertures. The upper chamber isolated the oocyte's upper domus and maintained it under clamp. The middle chamber provided a guard shield by clamping the middle part of the oocyte to the same potential as the upper chamber. The bottom chamber injected current intracellularly, through the saponin-permeabilized part of the oocyte. External solution (mM): 120 Na-methanesulfonate (MeS), 2 K-MeS, 2 $Ca(MeS)_2$, 10 HEPES (pH=7.0). Internal solution (mM): 120 K-glutamate, 10 HEPES (pH=7.0). Intracellular micro-pipette solution (mM): 2700 Na-MeS, 10 NaCl. Low access resistance to the oocyte interior was obtained by permeabilizing the oocyte with 0.1% saponin carried by the internal solution. In VCF experiments (set-up and staining procedures described below), holding potential was –80mV. All pulses were preceded by a 800ms pre-pulse to –120mV. 100-ms pulses were used (–120…160mV, ΔV=20mV) with inter-pulse duration of 10 s. Current was filtered to 5 kHz using the Dagan on-board low-pass Bessel filter. Data were acquired at 25 kHz using a Digidata 1550B1 digitizer and pClamp v.11 software (Molecular Devices).

Voltage clamp fluorometry (VCF): VCF was performed by adding epifluorescence and light detection capability to the COVG system (12, 13). Prior to mounting on the COVG apparatus, oocytes were stained at room temperature for 5 minutes with 20 µM MTS-TAMRA fluorophore in a depolarizing solution (in mM: 120 K-MeS, 2 $Ca(MeS)_2$, 10 HEPES, pH=7.0), in the dark, to label the introduced Cys (A291C). The oocytes were then rinsed in dye-free SOS prior to being mounted in the recording chamber. Fluorescence emission and ionic current were simultaneously measured from the same area of membrane isolated by the top chamber. The same electrophysiological apparatus and solutions were used as above. The optical setup consisted of an Olympus BX51WI upright microscope with filters (Semrock Brightline) appropriate for rhodamine excitation and emission wavelengths (exciter: FF01-531/40-25; dichroic: FF562-Di02-25x36; emitter: FF01-593/40-25). The light source was a 530 nm, 170 mW LED (M530L3-C1,

Thorlabs) driven by a Cyclops LED driver (Open Ephys). The objective (Olympus LUMPLANFL, 40XW, water immersion) had a numerical aperture of 0.8 and a working distance of 3.3 mm, which left sufficient room for the insertion of the microelectrode while fully covering the oocyte domus exposed in the external recording chamber. The emission light from the camera port was focused on a Si photodiode (SM05PD3A, Thorlabs) connected to a current amplifier (DLPCA-200, FEMTO). Its output was filtered to 5 kHz using an in-line low-pass Bessel filter (LPF-8, Warner Instruments) before being acquired at 25 kHz (Digidata 1550B1 and pClamp v.11, Molecular Devices).

Data analysis and modeling

Electrophysiological data analysis: All electrophysiological data analysis was performed by least squares fitting in Microsoft Excel. In experiments with just K_V1.2 subunits, steady-state activation was calculated by fitting the macroscopic conductance to a Boltzmann distribution:

$$G = \frac{G_{\mathrm{total}}}{1+\exp\left[\frac{z\mathrm{F}}{\mathrm{R}T}\left(V_{0.5}-V_{\mathrm{m}}\right)\right]} \qquad [1]$$

where V_m was the membrane potential; $V_{0.5}$ was the half-activation potential; z was the effective valence; F and R the Faraday and Gas constants, respectively; T was temperature (294 K). The maximal macroscopic conductance, G, was calculated by dividing the current (I_m) by the driving force:

$$G = \frac{I_{\mathrm{m}}}{V_{\mathrm{m}}-E_{\mathrm{K}}} \qquad [2]$$

where E_K was the equilibrium potential for potassium. In TEVC experiments, the V_m reported by OpusXpress at the same time as the sampled I_m was used.

In experiments with K_V1.4 subunits, peak current was used. The G-V curve could be better accounted-for with the sum of two Boltzmann distributions:

$$G = G_{\mathrm{total}}\sum_{i=1}^{2}\frac{w_i}{1+\exp\left[\frac{z_i\mathrm{F}}{\mathrm{R}T}\left(V_{0.5,i}-V_{\mathrm{m}}\right)\right]} \qquad [3]$$

where w is the fractional contribution, or weight, of each component, and $w_1=1-w_2$.

The voltage dependence of fluorescence deflections (ΔF; *i.e.*, VSD activation) was estimated by fitting ΔF to a Boltzmann distribution:

$$\Delta F = \frac{\Delta F_{\mathrm{max}}-\Delta F_{\mathrm{min}}}{1+\exp\left[\frac{z\mathrm{F}}{\mathrm{R}T}\left(V_{0.5}-V_{\mathrm{m}}\right)\right]}+\Delta F_{\mathrm{min}} \qquad [4]$$

where ΔF_{max} and ΔF_{min} were the maximal and minimal ΔF asymptotes, respectively.

Binomial models of heteromeric channel configurations: The probability that a tetrameric channel with specific subunit composition will form was calculated starting with the standard binomial distribution:

$$f\left(n,k,p\right)=\binom{n}{k}p^k\left(1-p\right)^{n-k} \qquad [5]$$

where n is the number of trials (always 4, for the obligate tetrameric K_V channels); k is the number of "successes", *i.e.*, $K_V1.2$(WT) or $K_V1.4$ subunits in the tetramer (k=4 for WT homo-tetramers, k=3 for 3:1 WT:F233S hetero-tetramers, and so on); p is the probability to encounter a $K_V1.2$(WT) or $K_V1.4$ subunit during tetramerization, given by:

$$p = \frac{1}{m+1} \qquad [6]$$

where m is the relative molar proportion of injected $K_V1.2$(F233S) cRNA, ranging from 0 to 8.

In these experiments (Figs. 5A, 7A), the molar proportion of $K_V1.2$(F233S) cRNA increased, while the amount of $K_V1.2$(WT) or $K_V1.4$ cRNA was kept stable. Therefore, more tetramers could potentially be made with higher F233S proportions due to the increased availability of K_V subunits. For this reason, the frequency of tetramers (f, eq.[5]) was scaled by the relative amount of cRNA:

$$g = f\left(n,k,p\right)\cdot(m+1) \qquad [7]$$

The black curves in the model (Figs. 5A, 7A) represent g with k=4; the green curves are the sum of g with k=4 and k=3; and so on.

Statistics: All comparative statistics (*e.g.*, "relative G_{total}" or "$\Delta V_{0.5}$") were performed among oocytes or COS-7 cells from the same block of experiments (same batch / passage number, RNA injection / DNA transfection, and experiment day). All significance tests were two-tailed Student's *t*-tests. Errors are SEM or 95% confidence intervals (C.I.), as stated in the figure legends.

Supplementary Figures

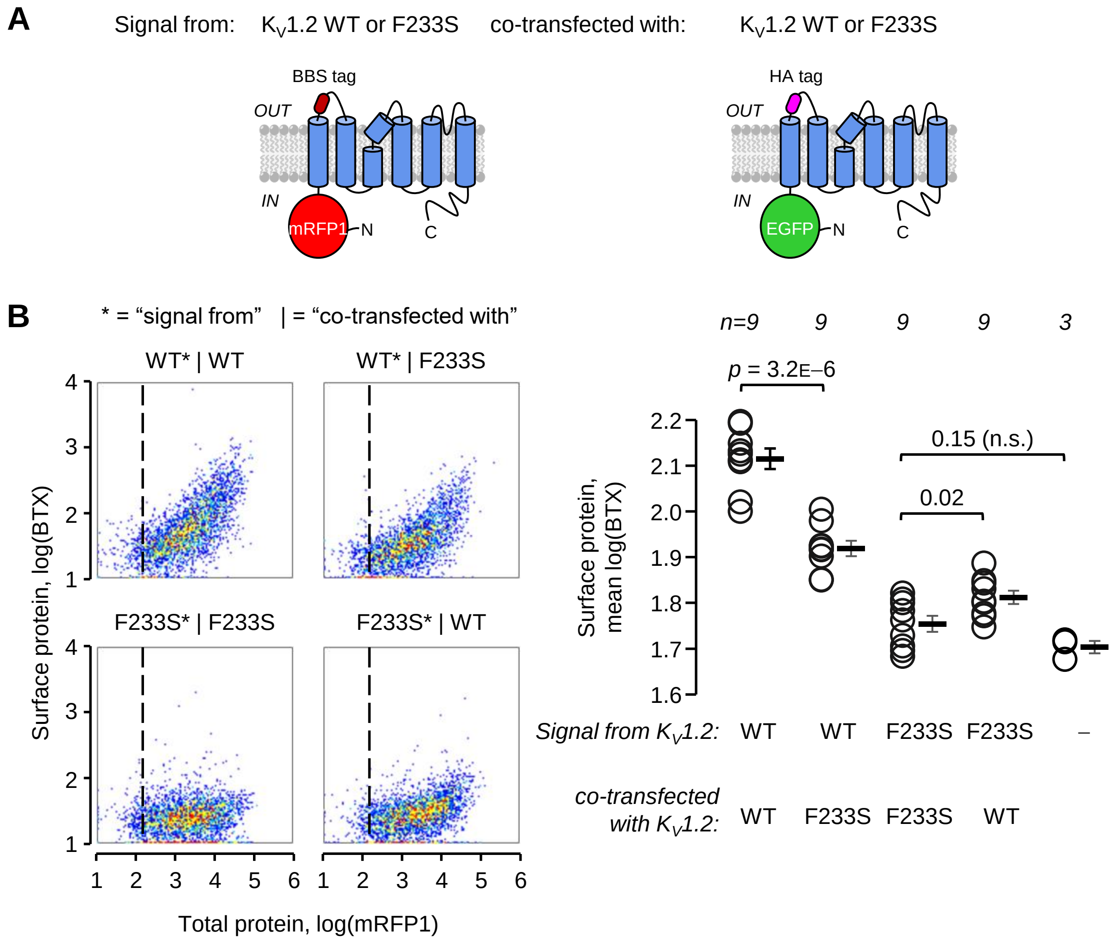

Fig. S1: Kv1.2(F233S) subunits sequester, and are concomitantly rescued by, Kv1.2(WT): the "flipped construct" experiment. Same dataset as in the experiment in Fig.4, showing the surface signals from BBS constructs. **(A)** Constructs used to evaluate Kv1.2 cell surface trafficking. Each construct represents one allele. (i) Kv1.2 with N-terminally fused mRFP1, reporting total protein production; and an extracellular bungarotoxin binding-site (BBS) tag, co-transfected with (ii) Kv1.2 with N-terminally fused EGFP, reporting total protein production; and an extracellular hemagglutinin (HA) tag. **(B)** Representative cell density plots of log(mRFP1) (total protein) against log(BTX) (cell surface expression) from cells positive for EGFP signal. WT*: mRFP1-Kv1.2(BBS); WT: EGFP-Kv1.2(HA); F233S*: mRFP1-Kv1.2(BBS,F233S); F233S: EGFP-Kv1.2(HA,F233S). The vertical dashed line separates negative (left) and positive (right) mRFP1 cells among the plotted live, EGFP-positive cells. Percentage of cells with positive surface (BTX) signal (normalized to WT*|WT, *i.e.*, 100±4.7%): WT*|F233S: 42±3.4 %; F233S*|WT: 13±2.2 %; F233S*|F233S: 5.5±1.1 %. Mean surface protein intensity (log(BTX)) from live, EGFP- and mRFP1-positive cells. Error bars are ±SEM. n.s.= not significant.

A

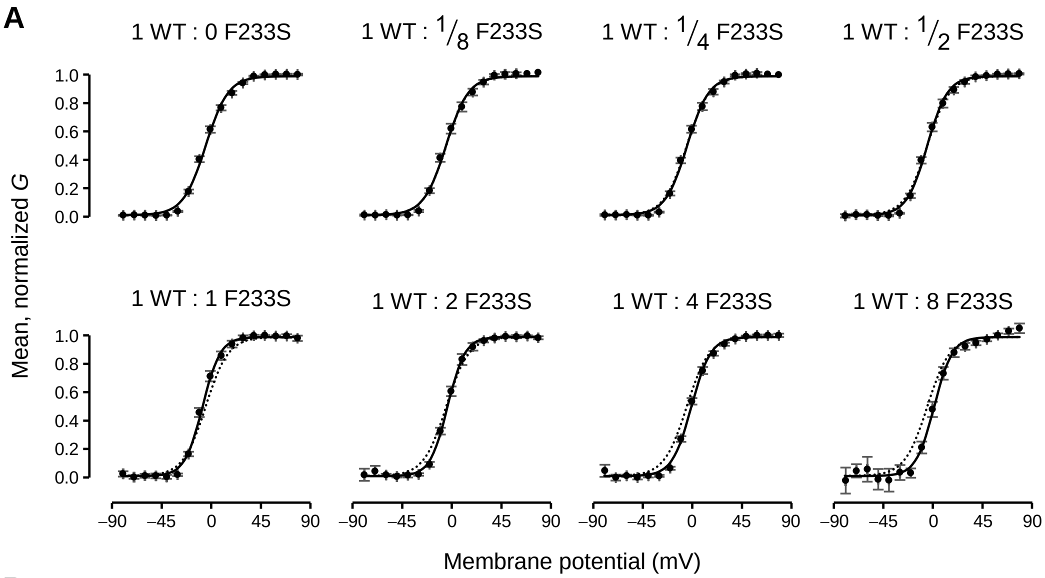

B

F233S molar proportion	0	1/8	1/4	1/2	1	2	4	8
G_{total} (nS)	67±12	92±23	72±15	32±6.8	19±6.0	15±4.6	10±1.7	2.7±0.5
$V_{0.5}$ (mV)	−4.9±0.9	−5.2±1.4	−4.8±1.0	−5.1±1.1	−8.3±1.3	−3.7±1.3	−0.8±0.9	1.1±1.8
z (e^0)	2.5±0.1	2.5±0.2	2.6±0.2	2.8±0.2	3.3±0.3	3.3±0.3	2.9±0.1	3.2±0.3
fold change in G_{total}	1.0±0.1	1.0±0.2	0.89±0.21	0.48±0.09	0.31±0.07	0.22±0.07	0.14±0.03	0.033±0.007
$\Delta V_{0.5}$ (mV)	0.0±0.6	−1.5±1.2	−1.0±0.8	−1.0±1.1	−2.3±1.2	−0.2±1.1	3.0±1.0	4.8±1.7
n	47	18	18	23	18	24	23	18

C

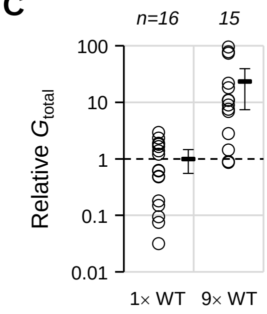

D

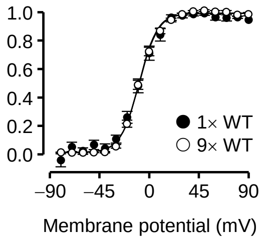

E

K_V1.2(WT) relative amount	1×	9×
G_{total} (nS)	8.5±4.2	190±120
$V_{0.5}$ (mV)	−11±2.9	−7.4±2.1
z (e^0)	3.3±0.51	2.7±0.35
fold change in G_{total}	1.0±0.45	23±16
$\Delta V_{0.5}$ (mV)	0.0±2.9	3.8±2.1

Fig. S2: Currents from oocytes injected with K_V1.2(WT) and increasing proportions of K_V1.2(F233S) exhibit similar voltage dependence. (A) Mean, normalized conductance (filled circles) and Boltzmann distribution fits (solid line) from the experiments in Fig.5A. The dotted line is the Boltzmann fit of 1 WT : 0 FS (top-left). **(B)** Boltzmann distribution parameters (see eq.1) for the fits in panel A. **(C)** Oocytes were injected with 9-fold K_V1.2(WT) cRNA, to evaluate oocyte capacity to process this amount of cRNA. Their macroscopic conductance was 23±8.2-fold

relative to oocytes expressing 1× RNA (*i.e.*, 0.5ng/oocyte). This amount is not statistically significant from a sample with mean=9 (*i.e.*, 9-fold increase in conductance) and the same standard deviation and number of observations (p=0.21). This experiment was performed under COVG, to handle the increased conductance, up to 730 μS, with better voltage control; in addition, current is recorded from 20% of the oocyte surface area (12), further decreasing macroscopic currents that could generate clamp errors. It shows that the conductance observed in the presence of increasing F233S cRNA (Figs.5A & 7A) was not under-estimated due to potential oocyte translation issues. **(D)** Voltage dependence of 1× and 9× WT RNA condition. **(E)** Parameters for the analysis in panes C and D. Errors are 95% C.I..

A

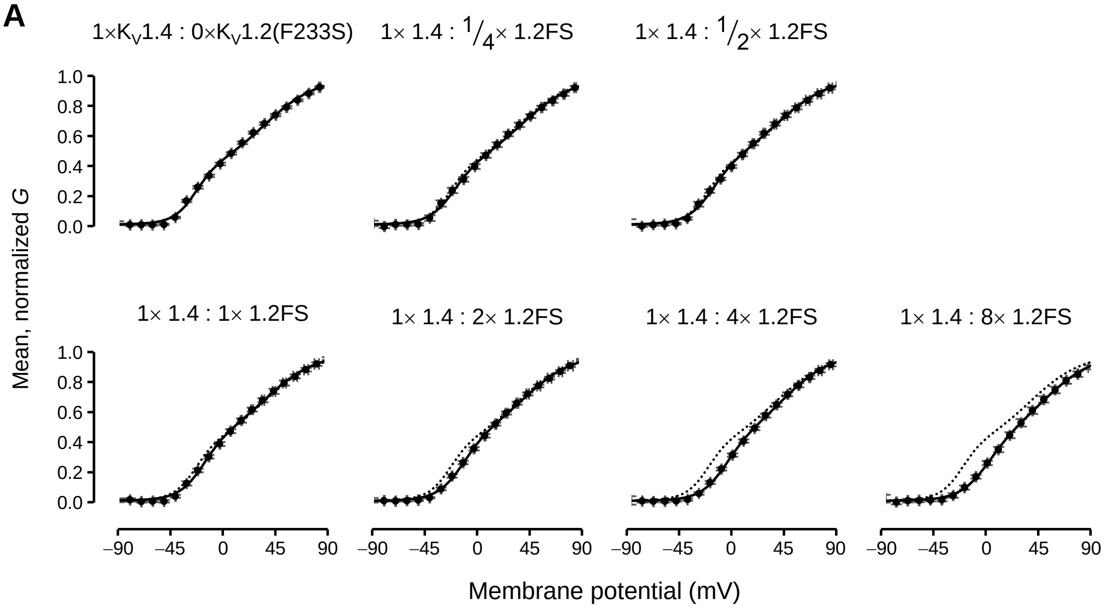

B

F233S molar proportion	0	1/4	1/2	1	2	4	8
G_{total} (nS)	28±4.0	26±6.4	39±11	39±9	49±8	28±4	15±3.4
$V_{0.5,\,1}$ (mV)	–23±1.0	–20±2.5	–19±1.2	–16±1.3	–11±1.3	–6.9±1.1	–0.5±2.4
z_1 (e^0)	2.8±0.1	2.7±0.2	2.4±0.1	2.5±0.2	2.3±0.1	2.3±0.1	2.2±0.2
w_2	0.65±0.01	0.63±0.02	0.61±0.02	0.59±0.01	0.58±0.01	0.60±0.01	0.60±0.05
$V_{0.5,\,2}$ (mV)	38±1.5	41±3.0	40±2.0	41±2.7	45±1.8	46±2.0	52±4.5
z_2 (e^0)	1.1±0.02	1.1±0.04	1.1±0.04	1.2±0.04	1.2±0.02	1.2±0.03	1.2±0.05
$V_{0.5}$ (mV)	12±1.4	14±2.4	11±1.7	12±2.2	15±1.6	20±1.4	27±2.7
$V_{0.2}$ (mV)	–25±0.8	–22±2.1	–22±1.1	–20±1.3	–16±1.2	–11±1.0	–5.5±1.5
fold change in G_{total}	1.0±0.10	0.78±0.13	1.4±0.32	1.5±0.26	1.44±0.30	0.83±0.12	0.48±0.11
$\Delta V_{0.5}$ (mV)	0.0±1.2	2.7±1.7	–0.4±1.9	–0.3±2.0	3.3±1.7	8.0±1.4	16±2.5
$\Delta V_{0.2}$ (mV)	0.0±0.6	2.2±1.7	2.8±0.9	4.4±1.1	9.0±1.2	14±0.8	19±1.1
n	43	18	22	30	23	23	18

Fig. S3: Boltzmann parameters for K_V1.4 channels coexpressed with increasing amount of K_V1.2(F233S). (A) Mean, normalized conductance (filled circles) and Boltzmann distribution fits (solid line) from the experiments in Fig.7A,B. The dotted line is the Boltzmann fit of 1× K_V1.4 : 0× K_V1.2(F233S) (top-left). **(B)** Boltzmann distribution parameters (see eq.3) for the curves in panel A and Fig.7B. Errors are ±95% C.I.

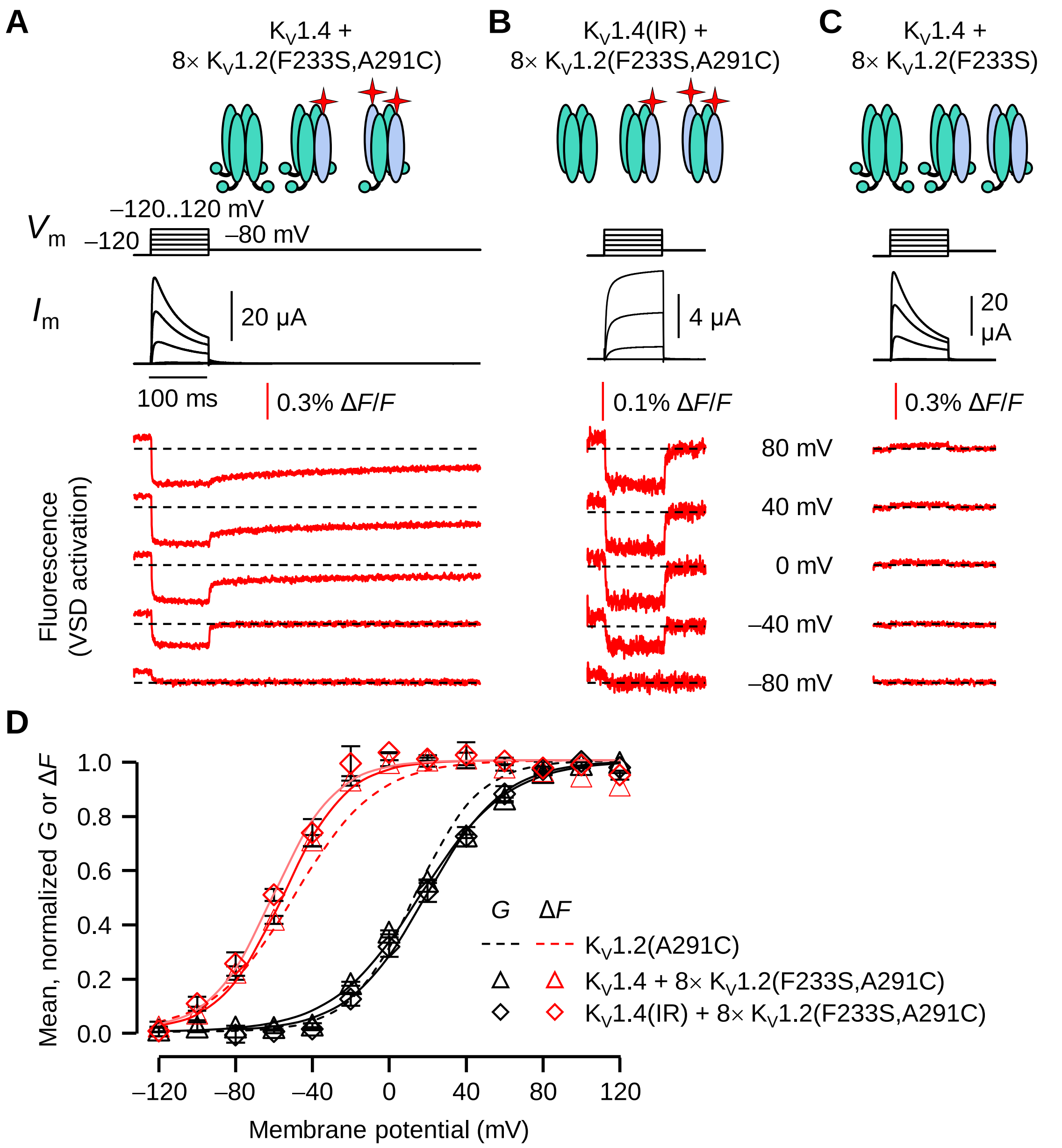

Fig. S4: Voltage-clamp fluorometry controls. (A) Voltage-clamp fluorometry (VCF) experiments on K_V1.2(F233S) subunits rescued by K_V1.4. Reproduced from Fig.8C, for reference. Note that VSD deactivation is very slow at potentials where channels open, and undergo N-terminal inactivation (charge immobilization). **(B)** VCF experiments on K_V1.2(F233S) subunits rescued by K_V1.4 lacking their inactivation particle (inactivation-removed, IR). Note that the channels do not inactivate, and the VSDs deactivate rapidly. **(C)** As in A, without A291C (negative fluorescence control). **(D)** Normalized macroscopic conductance (*G*, black) and VSD activation (Δ*F*, red), fit to Boltzmann distributions. K_V1.2(A291C) *G*: $V_{0.5}$=14±2.8 mV; z_{eff}=1.6±0.14 e^0; Δ*F*: $V_{0.5}$=–50±1.8 mV; z_{eff}=1.3±0.22 e^0; *n*=4. K_V1.2(F233S,A291C)/1.4 *G*: $V_{0.5}$=16±0.75 mV; z_{eff}=1.1±0.039 e^0; Δ*F*: $V_{0.5}$=–56±1.0 mV; z_{eff}=1.6±0.13 e^0; *n*=6. Errors are SEM. K_V1.2(F233S,A291C)/1.4(IR) *G*: $V_{0.5}$=18 [15,23] mV; z_{eff}=1.3 [1.3,1.2] e^0; Δ*F*: $V_{0.5}$=–61[–63,–58] mV; z_{eff}=1.6 [1.4,1.7] e^0; *n*=2. Error bars represent the two observations.

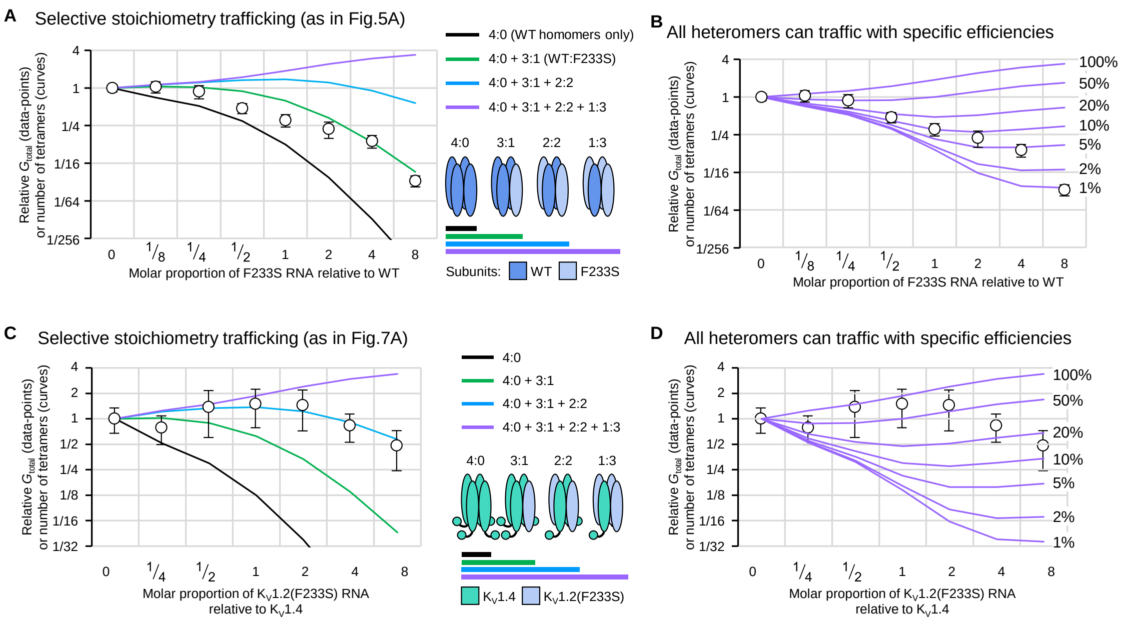

Fig. S5: Models with selective stoichiometry trafficking fit data better than those with equal heteromer trafficking efficiency. See Supplemental Discussion. **(A)** Reproduced from Fig.5A, the model shows that the trafficking of only 3WT:1F233S heterotetramers (as well as WT homo-tetramers) account for the data very well (green curve). **(B)** Same data as in A, but overlaid with models predicting equal trafficking efficiency for all hetero-tetramers, as specified. The curve with 100% efficiency is the same as the purple curve in A. The trafficking of WT homomers was always 100%. **(C,D)** as in A and B, for the $K_V1.4$ experiment (Fig.7A). Note that none of the curves in B and D describe the data as well as curves in A and C. While some do approximate the data (2% in B, and 20% efficiency in D), they contradict other experimental findings. Specifically, very low heteromeric trafficking efficiency in B contradicts the strong fluorescence signals showing F233S subunit rescue in immunocytochemistry (Figs. 4, S1) and VCF (Fig.8B) experiments. The 20% efficiency in D contradicts the ≈100% trafficking efficiency of $K_V1.4$-$K_V1.2$(F233S) concatemers in Fig.7D,E.

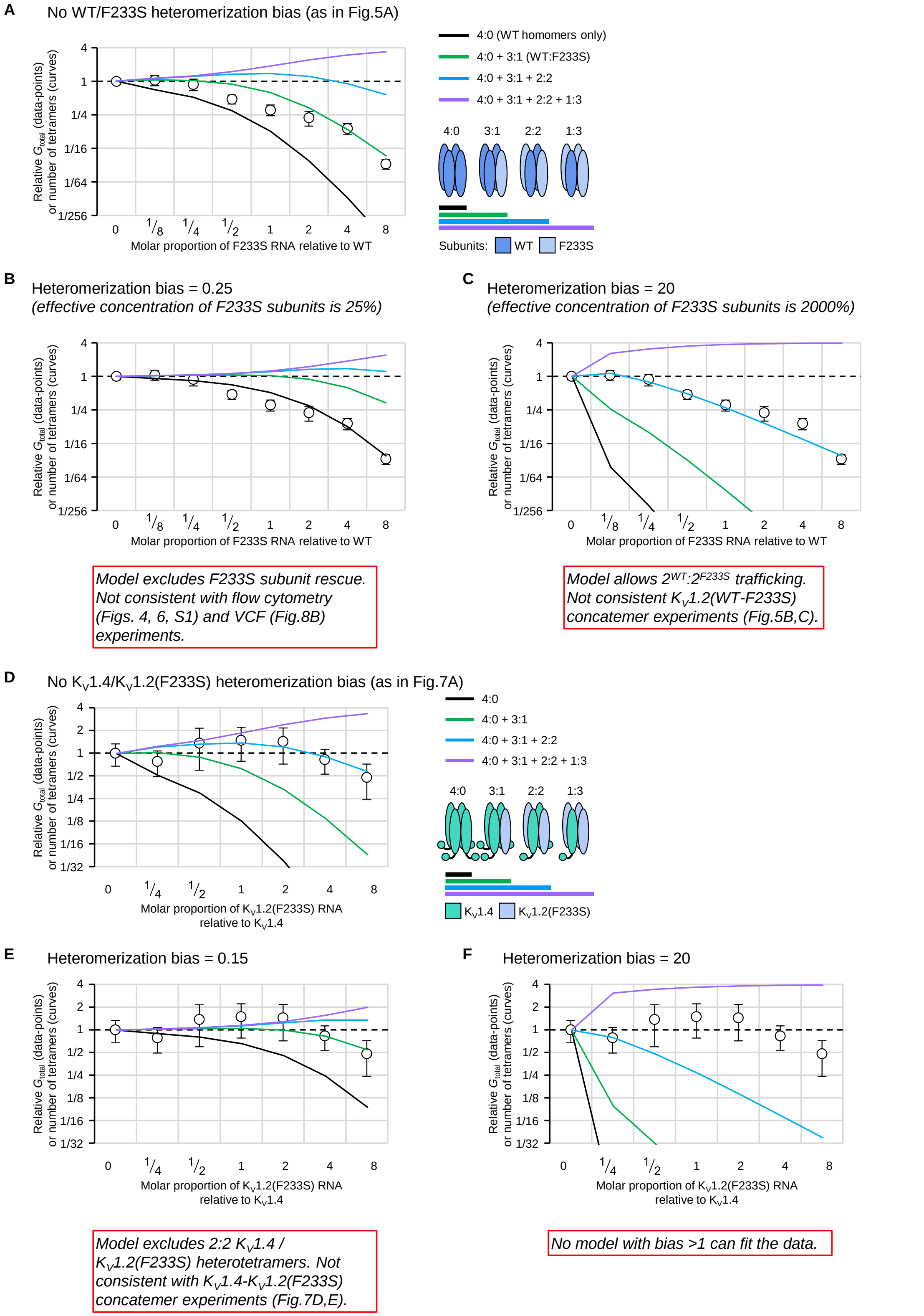

Fig. S6: Bias for, or against, heteromeric assembly is not consistent with experimental data. Tetramerization models incorporating negative (<1) or positive (>1) bias for heteromeric assembly between $K_V1.2$(F233S) and wild-type $K_V1.2$ **(A-C)** or $K_V1.4$ **(D-F)**. See Supplemental Discussion.

Table S1: Screened genes, and percentage of their coding region covered at a minimum of 10 reads

Gene	%	Gene	%	Gene	%	Gene	%	Gene	%	Gene	%	Gene	%	Gene	%
ABAT	99.99	*CARS2*	96.88	*DOCK7*	99.99	*GLRA1*	100	*KCTD7*	100	*PGAP2*	100	*SCARB2*	100	*STRADA*	99.63
ADGRG1	100	*CASK*	99.96	*DPAGT1*	100	*GLUD1*	98.14	*KPTN*	100	*PHGDH*	100	*SCN1A*	100	*STX1B*	100
ADPRHL2	100	*CASR*	100	*DYNC1H1*	100	*GNAO1*	100	*LGI1*	99.98	*PIGA*	98.54	*SCN1B*	99.77	*STXBP1*	100
ADSL	100	*CDKL5*	99.87	*DYRK1A*	100	*GNB1*	100	*LIAS*	100	*PIGG*	99.21	*SCN2A*	99.91	*SUOX*	100
AFG3L2	99.05	*CHD2*	100	*EARS2*	100	*GOSR2*	99.98	*MBD5*	100	*PIGN*	99.76	*SCN3A*	100	*SYN1*	99.81
AKT3	99.98	*CHRNA2*	100	*EEF1A2*	100	*GPHN*	100	*MBOAT7*	100	*PIGO*	100	*SCN8A*	100	*SYNGAP1*	94.64
ALDH5A1	100	*CHRNA4*	99.88	*EFHC1*	100	*GRIN1*	100	*MECP2*	99.98	*PIGT*	100	*SEPSECS*	100	*SYNJ1*	99.98
ALDH7A1	99.11	*CHRNB2*	100	*EHMT1*	97.03	*GRIN2A*	100	*MEF2C*	99.63	*PIGV*	100	*SERPINI1*	100	*SZT2*	100
ALG11	100	*CLCN4*	99.88	*EPM2A*	92.06	*GRIN2B*	99.91	*MFSD8*	100	*PLCB1*	100	*SHH*	100	*TBC1D24*	100
ALG13	99.78	*CLN3*	100	*EXOSC3*	98.74	*GRIN2D*	99.1	*MLC1*	100	*PLPBP*	100	*SIK1*	100	*TBCK*	99.92
ALPL	100	*CLN5*	100	*FARS2*	100	*HCN1*	100	*MOCS1*	99.52	*PNKP*	99.99	*SIX3*	100	*TCF4*	100
AMT	100	*CLN6*	99.98	*FGF12*	100	*HECW2*	99.99	*MOCS2*	100	*PNPO*	100	*SLC12A5*	100	*TPP1*	100
AP3B2	99.67	*CLN8*	100	*FLNA*	100	*HNRNPU*	100	*NARS2*	94.12	*POLG*	100	*SLC13A5*	100	*TRAK1*	99.23
ARFGEF2	100	*CLTC*	100	*FOLR1*	100	*HSD17B10*	100	*NDE1*	100	*PPP3CA*	99.98	*SLC19A3*	99.97	*TRIM8*	100
ARHGEF9	98.69	*CNKSR2*	99.9	*FOXG1*	97.47	*IER3IP1*	99.97	*NECAP1*	100	*PPT1*	100	*SLC1A2*	100	*TRPM6*	100
ARX	97.86	*CNTNAP2*	99.98	*FRRS1L*	94.03	*IQSEC2*	98.52	*NEDD4L*	99.99	*PRICKLE1*	100	*SLC25A12*	100	*TSC1*	99.99
ASAH1	99.97	*COL4A1*	99.96	*GABBR2*	99.89	*IRF2BPL*	99.97	*NEXMIF*	100	*PRRT2*	100	*SLC25A22*	100	*TSC2*	100
ASPM	99.89	*COQ4*	99.99	*GABRA1*	100	*ITPA*	97.33	*NGLY1*	100	*PRUNE1*	100	*SLC2A1*	100	*TSEN2*	98.67
ASXL3	99.93	*CPLX1*	100	*GABRA2*	99.99	*KANSL1*	99.95	*NHLRC1*	100	*PSAT1*	99.07	*SLC35A2*	98.86	*TSEN54*	96.82
ATP1A2	99.99	*CSTB*	100	*GABRB2*	100	***KCNA2***	**100**	*NPRL3*	100	*PSPH*	100	*SLC35A3*	99.97	*TUBA1A*	100
ATP1A3	100	*CTSD*	100	*GABRB3*	99.94	*KCNB1*	100	*OPHN1*	99.93	*PTCH1*	100	*SLC6A1*	100	*TUBB2A*	99.96
BCKDK	100	*CUL4B*	99.95	*GABRG2*	99.23	*KCNC1*	100	*OTUD6B*	100	*PURA*	100	*SLC6A8*	99.99	*UBA5*	100
BRAT1	99.99	*DCX*	100	*GAMT*	99.99	*KCNJ10*	100	*PACS1*	100	*QARS*	100	*SLC9A6*	99.96	*UBE3A*	99.55
C12orf57	100	*DENND5A*	100	*GATM*	100	*KCNMA1*	100	*PACS2*	100	*RARS2*	98.55	*SMC1A*	99.9	*WDR45*	99.08
CACNA1A	99.61	*DEPDC5*	99.62	*GCSH*	99.67	*KCNQ2*	100	*PAFAH1B1*	99.73	*RELN*	100	*SNAP25*	100	*WDR62*	100
CACNA1D	100	*DIAPH1*	98.42	*GFAP*	99.9	*KCNQ3*	100	*PARS2*	100	*RFT1*	99.99	*SPATA5*	100	*WDR73*	100
CACNA2D2	99.64	*DNM1*	100	*GLDC*	99.56	*KCNT1*	99.86	*PCDH12*	100	*ROGDI*	99.93	*SPTAN1*	100	*WWOX*	100
CAD	100	*DNM1L*	99.99	*GLI2*	100	*KCNT2*	99.98	*PCDH19*	99.97	*RORA*	99.76	*ST3GAL5*	99.66	*ZEB2*	100

.

SI references

1. J. Lu, J. M. Robinson, D. Edwards, C. Deutsch, T1-T1 interactions occur in ER membranes while nascent Kv peptides are still attached to ribosomes. *Biochemistry* **40**, 10934-10946 (2001).
2. S. Richards *et al.*, Standards and guidelines for the interpretation of sequence variants: a joint consensus recommendation of the American College of Medical Genetics and Genomics and the Association for Molecular Pathology. *Genet Med* **17**, 405-424 (2015).
3. C. Gu, Y. N. Jan, L. Y. Jan, A conserved domain in axonal targeting of Kv1 (Shaker) voltage-gated potassium channels. *Science* **301**, 646-649 (2003).
4. A. Pantazis *et al.*, Tracking the motion of the KV 1.2 voltage sensor reveals the molecular perturbations caused by a de novo mutation in a case of epilepsy. *J Physiol* **598**, 5245-5269 (2020).
5. K. Nakahira, G. Shi, K. J. Rhodes, J. S. Trimmer, Selective interaction of voltage-gated K+ channel beta-subunits with alpha-subunits. *J Biol Chem* **271**, 7084-7089 (1996).
6. S. Kondoh, K. Ishii, Y. Nakamura, N. Taira, A mammalian transient type K+ channel, rat Kv1.4, has two potential domains that could produce rapid inactivation. *J Biol Chem* **272**, 19333-19338 (1997).
7. A. Ray (2017) Development of novel concatemer technology using potassium channel (Kv1.1) homotetramer as a framework.
8. S. Akhtar, O. Shamotienko, M. Papakosta, F. Ali, J. O. Dolly, Characteristics of brain Kv1 channels tailored to mimic native counterparts by tandem linkage of alpha subunits: implications for K+ channelopathies. *J Biol Chem* **277**, 16376-16382 (2002).
9. B. Bourdin, E. Segura, M. P. Tetreault, S. Lesage, L. Parent, Determination of the Relative Cell Surface and Total Expression of Recombinant Ion Channels Using Flow Cytometry. *J Vis Exp* 10.3791/54732 (2016).
10. M. Taglialatela, L. Toro, E. Stefani, Novel voltage clamp to record small, fast currents from ion channels expressed in Xenopus oocytes. *Biophys J* **61**, 78-82 (1992).
11. E. Stefani, F. Bezanilla, Cut-open oocyte voltage-clamp technique. *Methods Enzymol* **293**, 300-318 (1998).
12. A. Pantazis, R. Olcese, "Cut-Open Oocyte Voltage-Clamp Technique" in Encyclopedia of Biophysics, G. Roberts, A. Watts, Eds. (Springer, Berlin, Heidelberg, 2019), 10.1007/978-3-642-35943-9_371-1, pp. 1-9.
13. C. S. Gandhi, R. Olcese, The voltage-clamp fluorometry technique. *Methods Mol Biol* **491**, 213-231 (2008).
